# Data for isolation and properties analysis of diastereomers of a mono-substituted phosphoryl guanidine trideoxyribonucleotide

**DOI:** 10.1016/j.dib.2019.104148

**Published:** 2019-06-20

**Authors:** Alexander A. Lomzov, Maxim S. Kupryushkin, Andrey V. Shernyukov, Mikhail D. Nekrasov, Ilya S. Dovydenko, Dmitry A. Stetsenko, Dmitrii V. Pyshnyi

**Affiliations:** a Institute of Chemical Biology and Fundamental Medicine, SB RAS, 8 Lavrentiev Avenue, Novosibirsk, 630090, Russia; b N.N. Vorozhtsov Novosibirsk Institute of Organic Chemistry, SB RAS, 9 Lavrentiev Avenue, Novosibirsk, 630090, Russia; c Novosibirsk State University, 2 Pirogova St., Novosibirsk 630090, Russia

**Keywords:** Phosphoryl guanidine oligonucleotide, RP-HPLC, Circular dichroism, NMR, Molecular dynamics, Diastereomer assignment phosphodiesterase digestion assay, Spatial structure

## Abstract

This article presents new data on the properties of the diastereomers of a mono-substituted phosphoryl guanidine trideoxyribonucleotides d(TpCp*A) [1,2]. The data include information on isolation, identification, treatment with snake venom phosphodiesterase and structural analysis by 1D and 2D NMR spectroscopy and restrained molecular dynamics analysis. The data can be used for preparation, analysis, application of phosphoryl guanidine oligonucleotide and for development of new nucleic acids derivatives. This data article is associated with the manuscript titled “Diastereomers of a mono-substituted phosphoryl guanidine trideoxyribonucleotide: isolation and properties” [1].

**Table:** Specifications Table

Subject area	*Chemistry, Physical Chemistry, Biology*
More specific subject area	*Biochemistry and physical chemistry of nucleic acids*
Type of data	*Table, graph, figure*
How data was acquired	*High pressure liquid chromatography, mass spectroscopy, Circular Dichroism spectroscopy, 1D and 2D NMR, Molecular dynamics simulations*
Data format	*Raw and analyzed data*
Experimental factors	Native trinucleotide d(TpCpA) and individual diastereomers of mono-substituted phosphoryl guanidine oligonucleotide d(TpCp*A) were purified and analyzed
Experimental features	Individual diastereomers of mono-substituted phosphoryl guanidine oligonucleotide d(TpCp*A) were purified by RP-HPLC using C18 sorbent and gradient of acetonitrile. MALDI-TOF MS analysis was conducted on Reflex III, Autoflex Speed with 3-hydroxypicolinic acid as a matrix. Temperature series of CD spectra were measured on a J-600 spectropolarimeter. NMR spectra were acquired on a Bruker Avance 600 MHz *spectrometer. MD simulation was performed using AMBER 14 MD modeling software with GPU accelerated code*
Data source location	*Institute of Chemical Biology and Fundamental Medicine of Siberian Branch of the Russian Academy of Sciences, 8 Lavrentiev Ave., Novosibirsk, 630090, Russian Federation* *NMR data were collected in N.N. Vorozhtsov Novosibirsk Institute of Organic Chemistry of Siberian Branch of the Russian Academy of Sciences, 9 Lavrentiev Ave., Novosibirsk, 630090, Russian Federation*
Data accessibility	*Data is with this article*
Related research article	*A. A. Lomzov, M. S. Kupryushkin, A. V. Shernyukov, M. D. Nekrasov, I. S. Dovydenko, D. A. Stetsenko, D. V. Pyshnyi, Diastereomers of a mono-substituted phosphoryl guanidine trideoxyribonucleotide: Isolation and properties, Biochem. Biophys. Res. Commun., 514,**2019**, 807–811**[1]**.*

**Table:** 

Value of the data • Data on the isolation, SVPDE digestion and identification of the mono-substituted phosphoryl guanidine oligonucleotide d(TpCp*A) diastereomers can be helpful for other researchers to analyze phosphate modified nucleic acids derivatives • The data can be used by other researchers with an interest in synthesis, purification and application of nucleic acid derivative and analogues • Our data contribute to the properties of phosphate-substituted oligonucleotides • This data could be useful for the researchers with an interest in biosensor development and biomedical application of nucleic acids

## 1 Data

Data reported here describe the features of diastereomers of a trideoxynucleotide 5′-TpCpA-3′ modified at the phosphate group near the 3′-end with a single 1,1,3,3-tetramethyl guanidine group revealed from studies by Revese phase HPLC (RP-HPLC) separation and analysis, SVPDE digestion, circular dichroism spectroscopy, 1D and 2D NMR analysis and restrained molecular dynamics simulation.

### 1.1 RP-HPLC analysis of oligonucleotides reaction mixture after synthesis

**Fig. 1:**
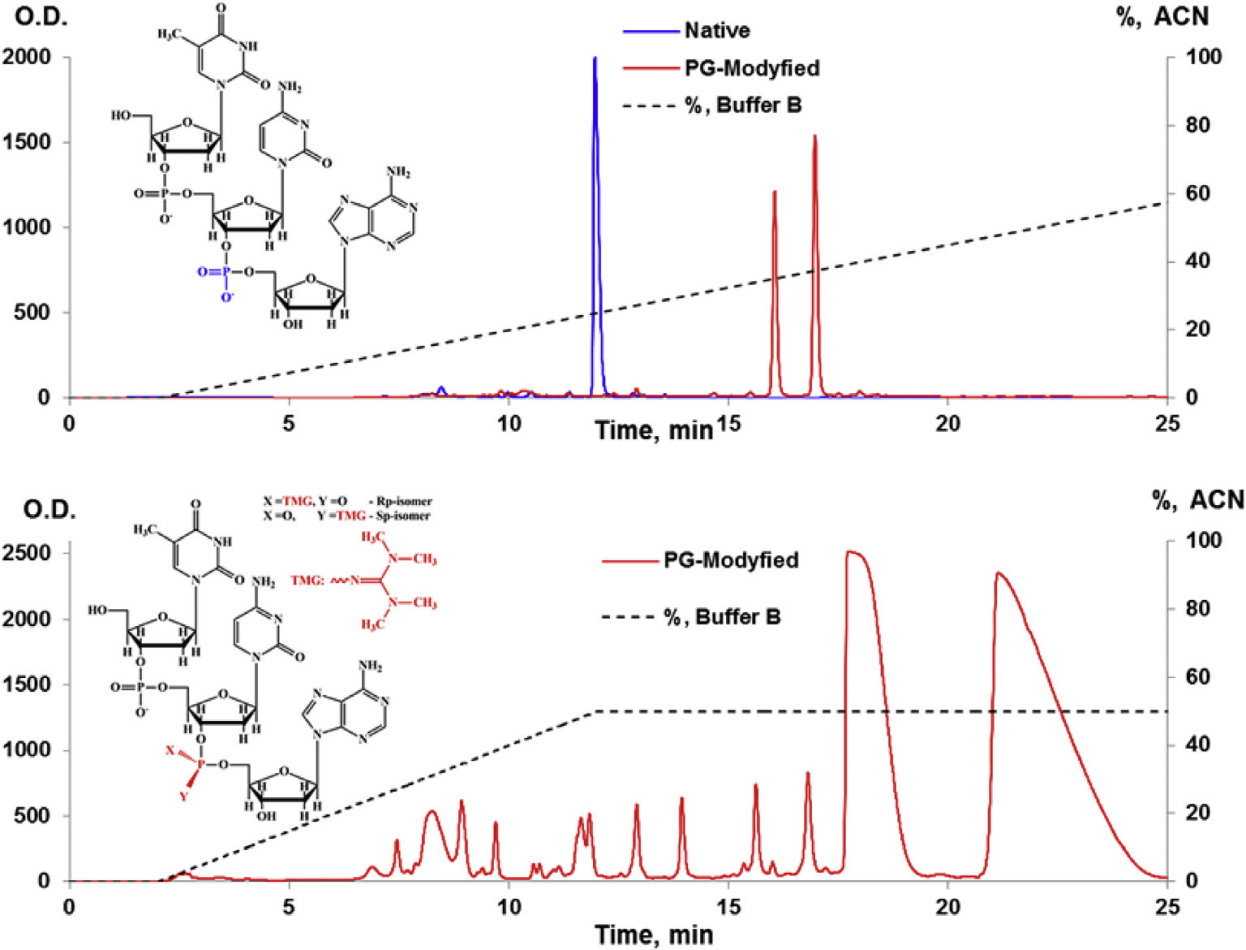
RP-HPLC analysis of reaction mixtures of native TpCpA (blue) and modified TpC*pA (red, * - position of modyfied phosphate) deoxyribotrinucleotides reaction mixture after detritilation. Upper chromatographic profiles are analytical and lower is preparative. Details of experiments see in section Material and methods.

Revese phase HPLC analysis of oligonucleotides were performed for reaction mixture of native d(TpCp*A) and mono-substituted phosphoryl guanidine (PG) oligonucleotides d(TpCp*A) after synthesis. Аnalytical and preparative chomatograms are shown in Fig. 1.

MALDI-TOF MS spectra of oligonucleotides.

**Fig. 2:**
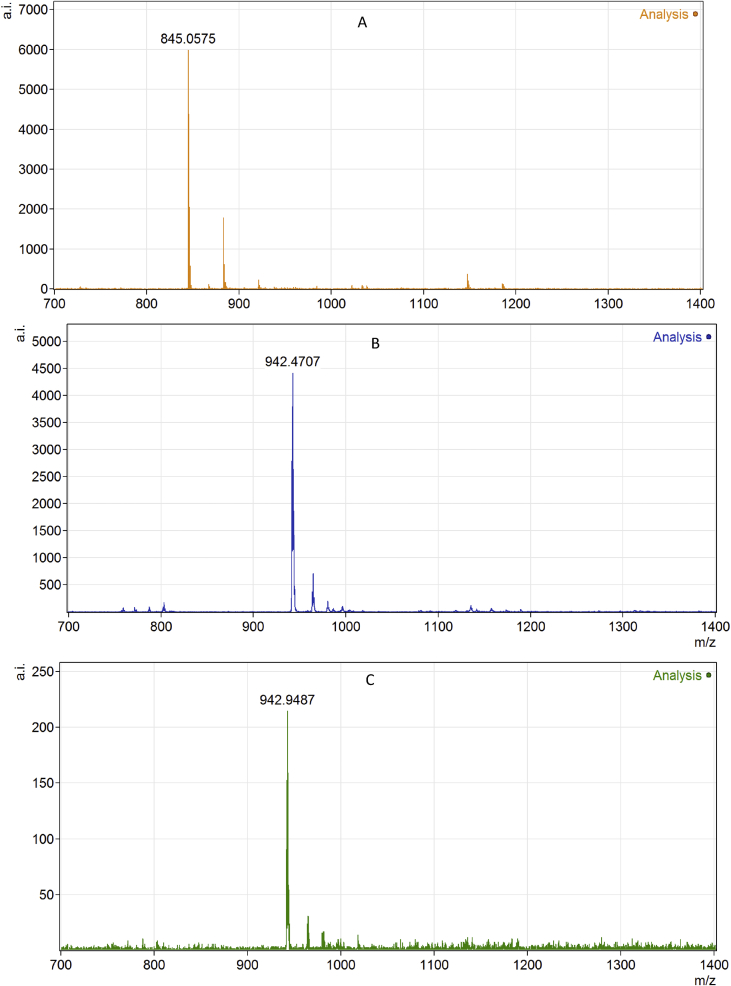
MALDI-TOF MS spectra of native (A) and PG-modified ‘fast’ (B) and ‘slow’ (C) trinucleotides.

Matrix-assisted laser desorption ionization – time of flight mass spectroscopy (MALDI-TOF MS) was conducted for the isolated by RP-HPLC samples of d(TpCpA) and diasteremers of d(TpCp*A) (Fig. 2).

## 1.2 RP-HPLC profiles of oligonucleotides after SVPDE digestion

**Fig. 3:**
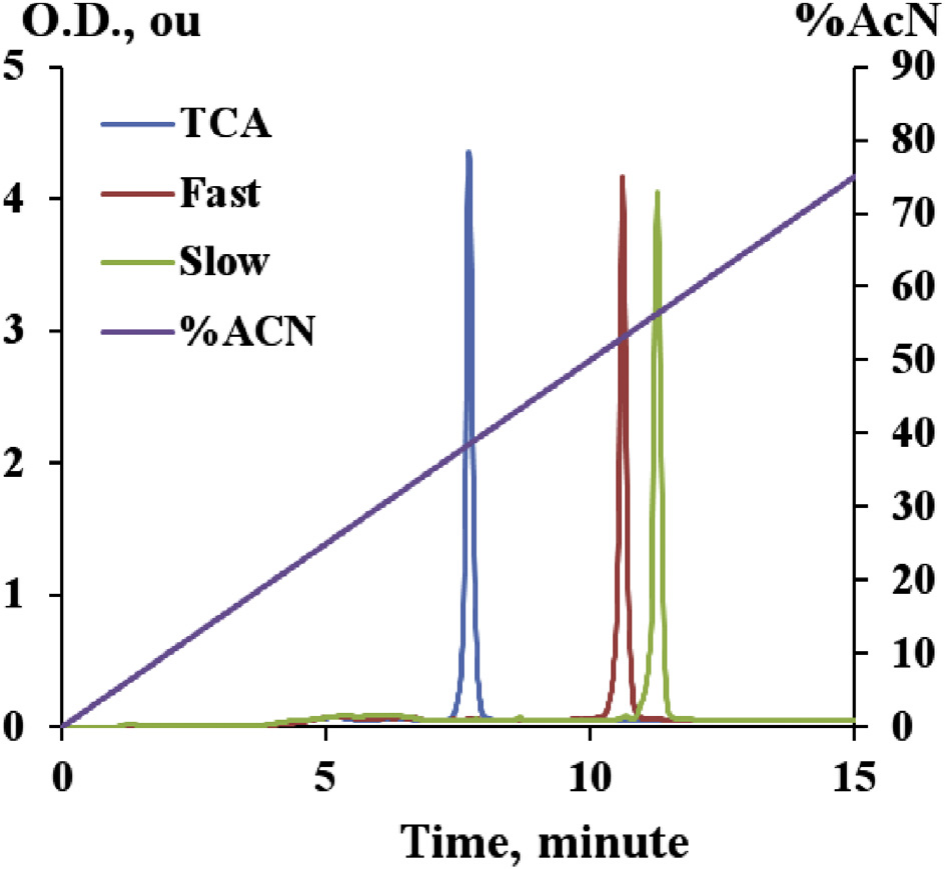
RP-HPLC profiles of TpCpA, TpCp*A(‘fast’) and TpCp*A (‘slow’) before SVPD digestion. Data on oligonucleote analysis after digestion shown in Fig. 2 in Ref. [1].

We treated native and mono-substituted oligonucleotides with snake venom phosphodiesterase (SVPDE) for 150 h. Three oligomers after digestion by SVPDE were analyzed by RP-HPLC (Fig. 3).

## 1.3 Circular dichroism spectra of oligonucleotides at high and low temperatures

**Fig. 4:**
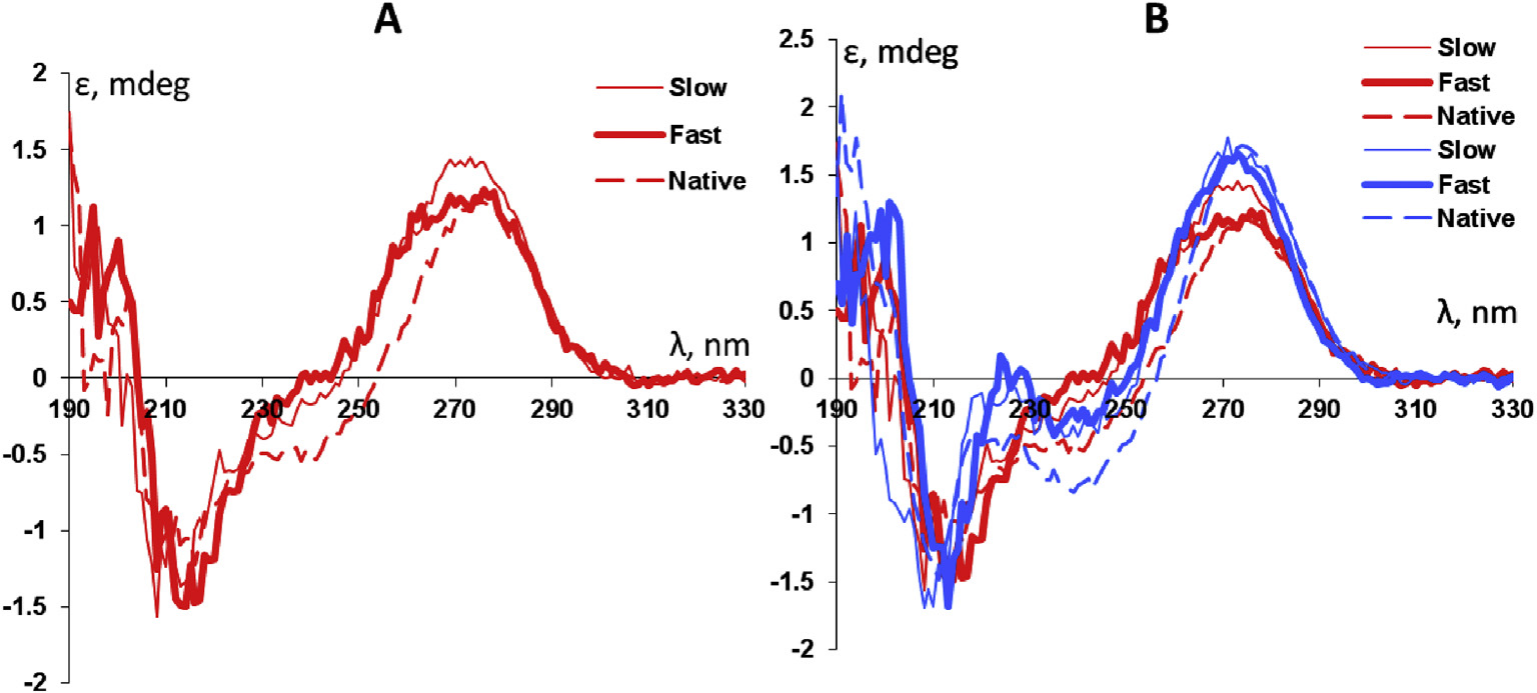
Circular dichroism spectra of native (dashed line), ‘fast’ (thick line) and ‘slow’ (thin line) trinucleotides at 95 °C (A) and comparison with 25 °C (B).

Circular dichroism spectra were used for chracterisation structure of native and modyfied oligonucleotides at low (25 °C) and high (95 °C) temperatures (Fig. 4).

## 1.4 NMR spectroscopy analysis of oligonucleotides

**Fig. 5:**
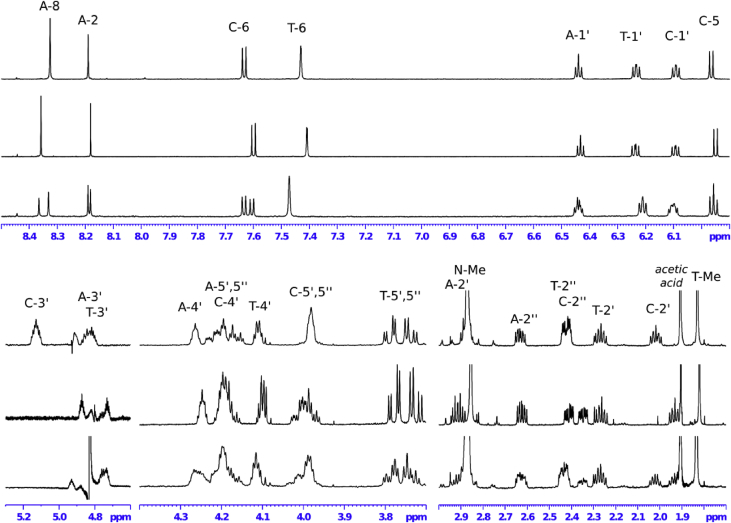
Fragments ^1^H NMR spectra (600 MHz, D_2_O). For each fragment ‘slow’ (top), ‘fast’ (middle) and their 1:1 mixture (down).

**Fig. 6:**
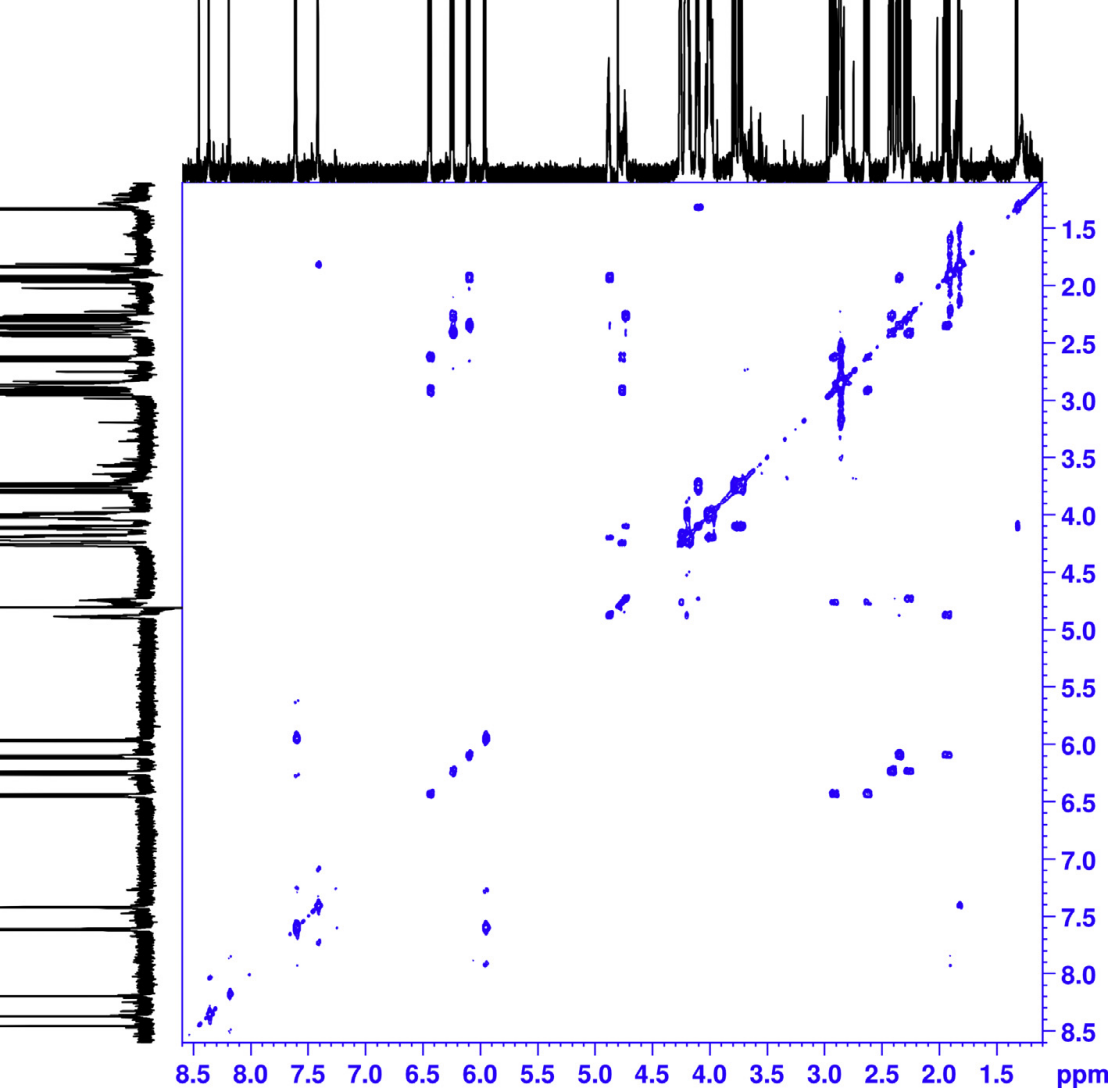
^1^H–^1^H COSY NMR spectra of the ‘fast’ isomer.

**Fig. 7:**
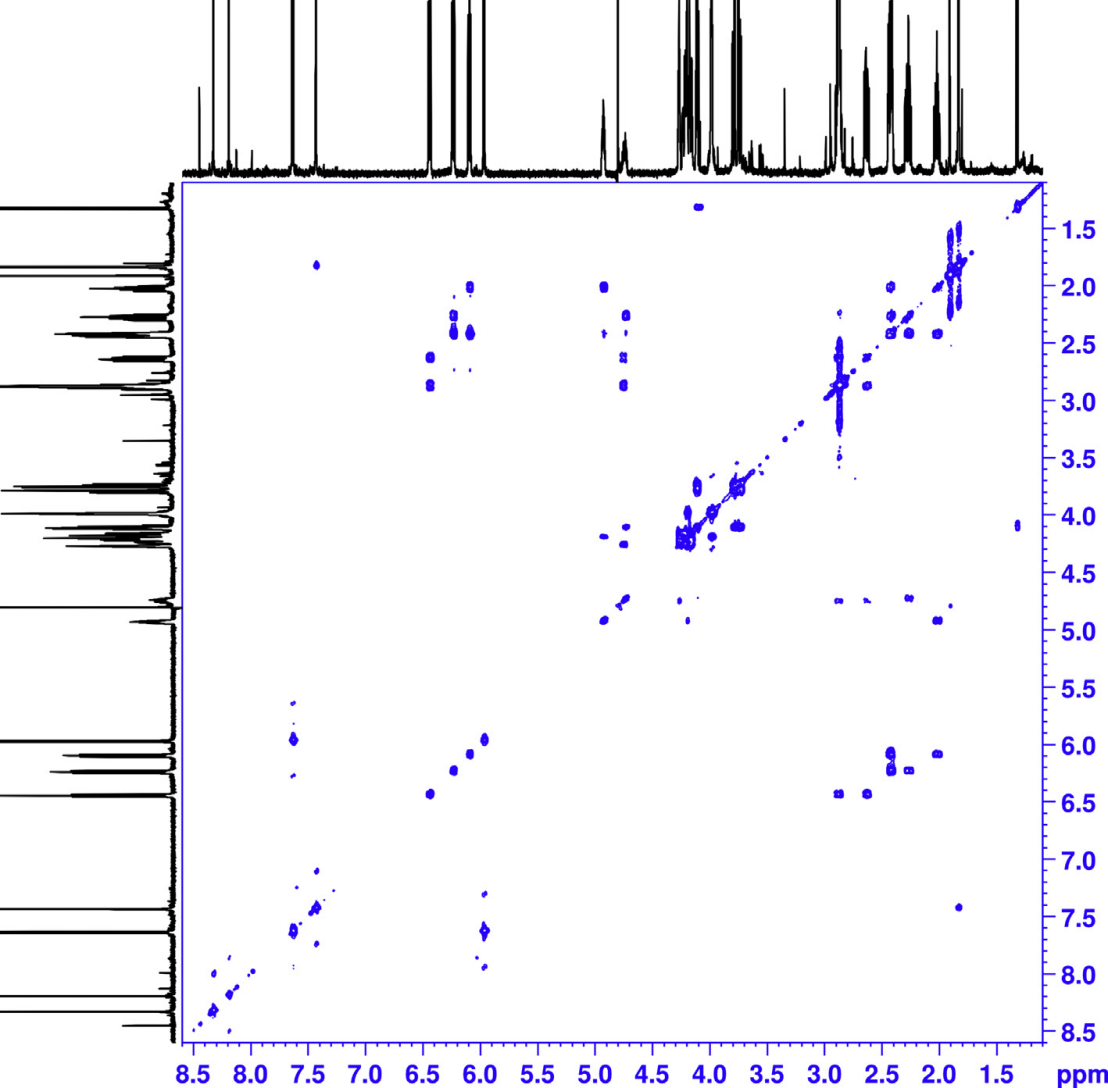
^1^H–^1^H COSY NMR spectra of the ‘slow’ isomer.

**Fig. 8:**
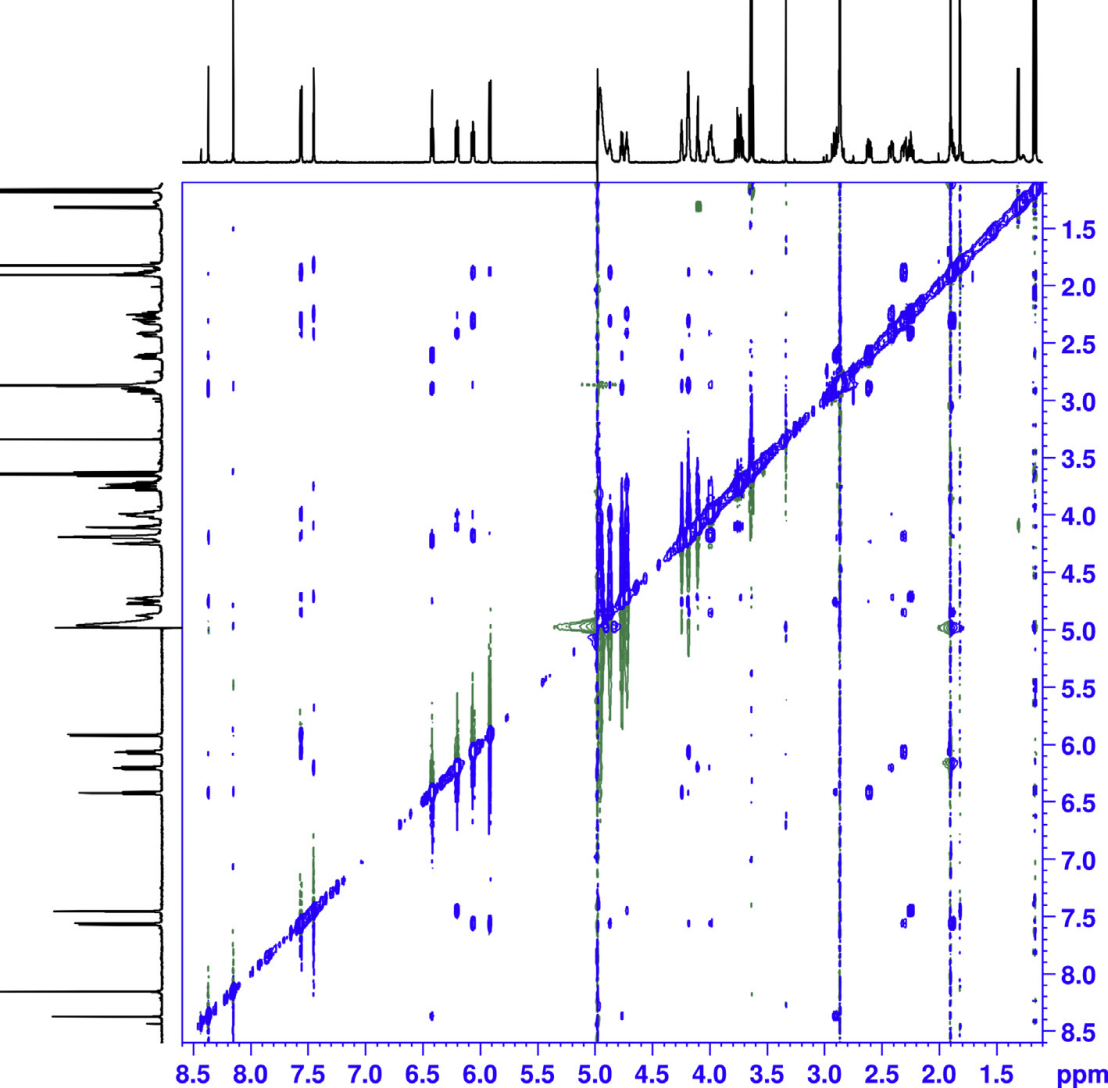
^1^H–^1^H NOESY NMR (mixing time 0.8s, T = 8.0 °C) spectra of the ‘fast’ isomer.

**Fig. 9:**
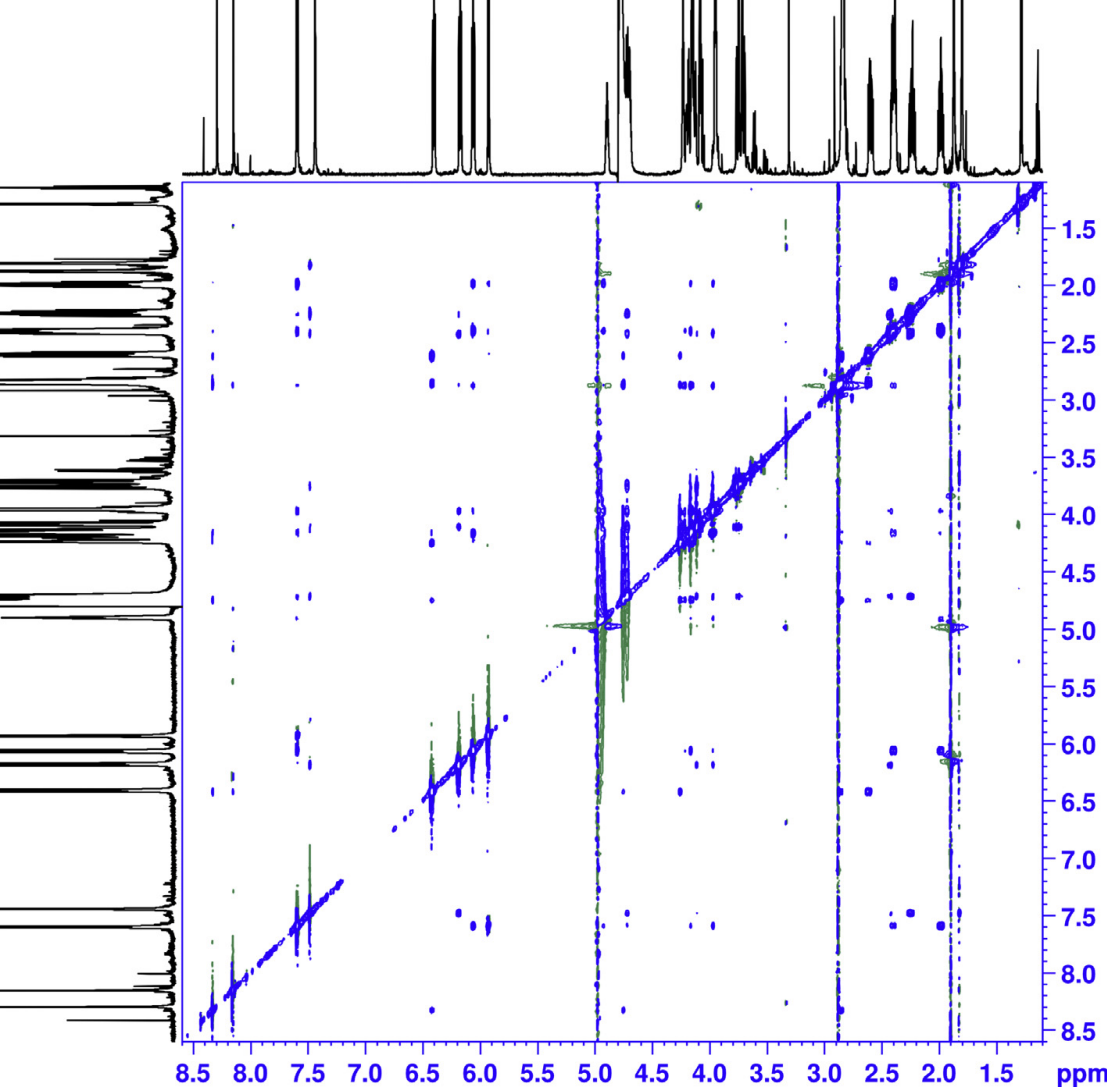
^1^H–^1^H NOESY NMR (mixing time 0.8s, T = 8.0 °C) spectra of the ‘slow’ isomer.

1D and 2D NMR spectroscopy experiments were performed for isolated mono-substituted phosphoryl guanidine oligonucleotides d(TpCp*A) and their mixture (Fig. 5, Fig. 6, Fig. 7, Fig. 8, Fig. 9, Table 1, Table 2, Table 3, Table 4, Table 5, Table 6, Table 7, Table 8, Table 9, Table 10).

**Table 1:** Chemical shits of ‘fast’ diastereomer, ppm.

	dT	dC	d(p*A)
**H1'**	6.25	6.10	6.44
**H2**	–	–	8.19
**H2'**	2.27	1.93	2.91
**H2''**	2.42	2.36	2.62
**H3'**	4.74	4.88	4.77
**H4'**	4.11	4.22	4.27
**H5**	–	5.96	–
**H5' & H5''**	3.79 и 3.73	4.04 и 4.00	4.18 и 4.20
**H6**	7.42	7.61	–
**H7**	1.83	–	–
**H8**	–	–	8.37
**C1'**	88.18	88.34	86.21
**C2**	161.56	159.84	155.53
**C2'**	40.24	40.48	41.20
**C3'**	78.12	78.55	73.09
**C4**	178.63	168.61	151.56
**C4'**	87.96	86.50	87.79
**C5**	114.49	99.16	121.36
**C5'**	63.96	67.43	68.20
**C6**	139.19	143.75	158.17
**C7**	15.50	–	–
**C8**	–	–	142.49
(CH_3_)_2_–N–CN-Cp*A	2.86		
(CH_3_)_2_–N–CN-Cp*A	42.38		
-C=N-Cp*A	165.88		
TpC	−0.82		
Cp*A	0.37

**Table 2:** Coupling constants^31^P–^13^C of ‘fast’ diastereomer, Hz.

	dT	dC		d(p*A)
	TpC	TpC	Cp*A	Cp*A
**C2'**	2.9	0	2.9	0
**C3'**	5.3	0	5.5	0
**C4'**	6.8	8.8	7.6	8.8
**C5'**	0	5.5	0	6.8
C=N-Cp*A = 7.5

**Table 3:** Chemical shits of ‘slow’ diastereomer, ppm.

	dT	dC	d(p*A)
**H1'**	6.23	6.09	6.44
**H2**	–	–	8.19
**H2'**	2.27	2.02	2.88
**H2''**	2.42	2.42	2.63
**H3'**	4.73	4.92	4.75
**H4'**	4.11	4.20	4.26
**H5**	–	5.96	–
**H5' & H5''**	3.74 и 3.79	3.98 и 3.99	4.17 и 4.22
**H6**	7.41	7.64	–
**H7**	1.82	–	–
**H8**	–	–	8.33
**C1'**	88.14	88.42	86.25
**C2**	160.69	159.89	155.52
**C2'**	40.22	40.7	41.27
**C3'**	78.11	78.64	73.20
**C4**	177.5	168.65	151.56
**C4'**	88.02	86.40	87.82
**C5**	114.45	99.1	121.40
**C5'**	63.94	68.10	67.29
**C6**	139.29	143.84	158.2
**C7**	15.37	–	–
**C8**	–	–	142.40
(CH_3_)_2_–N–CN-Cp*A	2.87		
(CH_3_)_2_–N–CN–C*pA	42.42		
-C=N-Cp*A	165.91		
TpC	−0.89		
Cp*A	0.21

**Table 4:** Coupling constants^31^P–^13^C of ‘slow’ diastereomer, Hz.

	dT	dC		d(p*A)
	TpC	TpC	Cp*A	Cp*A
**C2'**	2.8	0	2.6	0
**C3'**	5.4	0	6.5	0
**C4'**	6.6	8.5	6.9	8.9
**C5'**	0	5.1	0	5.8
C=N-Cp*A = 7.7

**Table 5:** Coupling constants ^1^H–^1^H, ^1^H–^31^P of adenosine monophosphate of ‘fast’ diastereomer, Hz.

	H1'	H2'	H2''	H3'	H4'	H5' & H5''	Cp*A
**H1'**	*	6.3	6.7	0	0	0	0
**H2'**	6.3	*	14.0	6.4	0	0	0
**H2''**	6.7	14.0	*	4.9	0	0	0
**H3'**	0	6.4	4.9	*	4.4	0	0
**H4'**	0	0	0	4.4	*	3.9	2.1
**H5' & H5''**	0	0	0	0	3.9	n.d..	n.d..
Cp*A	0	0	0	0	2.1	n.d..	*

**Table 6:** Coupling constants ^1^H–^1^H, ^1^H–^31^P of adenosine monophosphate of ‘slow’ diastereomer, Hz.

	H1'	H2'	H2''	H3'	H4'	H5' & H5''	Cp*A
**H1'**	*	6.5	6.5	0	0	0	0
**H2'**	6.5	*	14.0	6.4	0	0	0
**H2''**	6.5	14.0	*	4.8	0	0	0
**H3'**	0	6.4	4.8	*	4.3	0	0
**H4'**	0	0	0	4.3	*	3.9	2.1
**H5' & H5''**	0	0	0	0	3.9	11.5	4.9, 4.0
Cp*A	0	0	0	0	2.1	4.9, 4.0	*

**Table 7:** Coupling constants ^1^H–^1^H, ^1^H–^31^P of timidine of ‘fast’ diastereomer, Hz.

	H1'	H2'	H2''	H3'	H4'	H5' & H5''	H6	H7	TpC
**H1'**	*	8.1	6.1	0	0	0	0	0	0
**H2'**	8.1	*	14.1	6.3	0	0	0	0	0
**H2''**	6.1	14.1	*	3.0	0	0	0	0	0
**H3'**	0	6.3	3.0	*	3.2	0	0	0	7.0
**H4'**	0	0	0	3.2	*	3.5	0	0	0
**H5' & H5''**	0	0	0	0	3.5	12.5	0	0	0
**H6**	0	0	0	0	0	0	*	1.1	0
**H7**	0	0	0	0	0	0	1.1	*	0
**TpC**	0	0	0	7.0	0	0	0	0	*

**Table 8:** Coupling constants ^1^H–^1^H, ^1^H–^31^P of timidine of ‘slow’ diastereomer, Hz.

	H1'	H2'	H2''	H3'	H4'	H5' & H5''	H6	H7	TpC
**H1'**	*	8.0	6.1	0	0	0	0	0	0
**H2'**	8.0	*	14	6.2	0	0	0	0	0
**H2''**	6.1	14	*	3.0	0	0	0	0	0
**H3'**	0	6.2	3.0	*	3.2	0	0	0	7.0
**H4'**	0	0	0	3.2	*	3.5	0	0	0
**H5' & H5''**	0	0	0	0	3.5	12.5	0	0	0
**H6**	0	0	0	0	0	0	*	1.1	0
**H7**	0	0	0	0	0	0	1.1	*	0
**TpC**	0	0	0	7.0	0	0	0	0	*

**Table 9:** Coupling constants ^1^H–^1^H, ^1^H–^31^P of cytidine monophosphate of ‘fast’ diastereomer, Hz.

	H1'	H2'	H2''	H3'	H4'	H5' &H5''	H5	H6	TpC	Cp*A
**H1'**	*	8.0	6.0	0	0	0	0	0	0	0
**H2'**	8.0	*	14.2	6.3	0	0	0	0	0	0
**H2''**	6.0	14.2	*	2.8	0	0	0	0	0	0
**H3'**	0	6.3	2.8	*	2.7	0	0	0	0	7.0
**H4'**	0	0	0	2.7	*	2.8	0	0	n.d..	n.d..
**H5' & H5''**	0	0	0	0	2.8	11.6	0	0	4.8	0
**H5**	0	0	0	0	0	0	*	7.6	0	0
**H6**	0	0	0	0	0	0	7.6	*	0	0
**TpC**	0	0	0	0	n.d..	4.8	0	0	*	0
Cp*A	0	0	0	7.0	n.d..	0	0	0	0	*

**Table 10:** Coupling constants ^1^H–^1^H, ^1^H–^31^P of cytidine monophosphate of ‘slow’ diastereomer, Hz.

	H1'	H2'	H2''	H3'	H4'	H5' & H5''	H5	H6	TpC	Cp*A
**H1'**	*	7.9	6.0	0	0	0	0	0	0	0
**H2'**	7.9	*	14.2	6.3	0	0	0	0	0	0
**H2''**	6.0	14.2	*	2.8	0	0	0	0	0	0
**H3'**	0	6.3	2.8	*	2.8	0	0	0	0	7.0
**H4'**	0	0	0	2.8	*	3.4	0	0	n.d..	n.d..
**H5' & H5''**	0	0	0	0	3.4	11.7	0	0	4.2 4.6	0
**H5**	0	0	0	0	0	0	*	7.5	0	0
**H6**	0	0	0	0	0	0	7.5	*	0	0
**TpC**	0	0	0	0	n.d..	4.2, 4.6	0	0	*	0
Cp*A	0	0	0	7.0	n.d..	0	0	0	0	*

Assignment of the NMR signals.

## 1.5 Molecular dynamics simulation data analysis

**Fig. 10:**
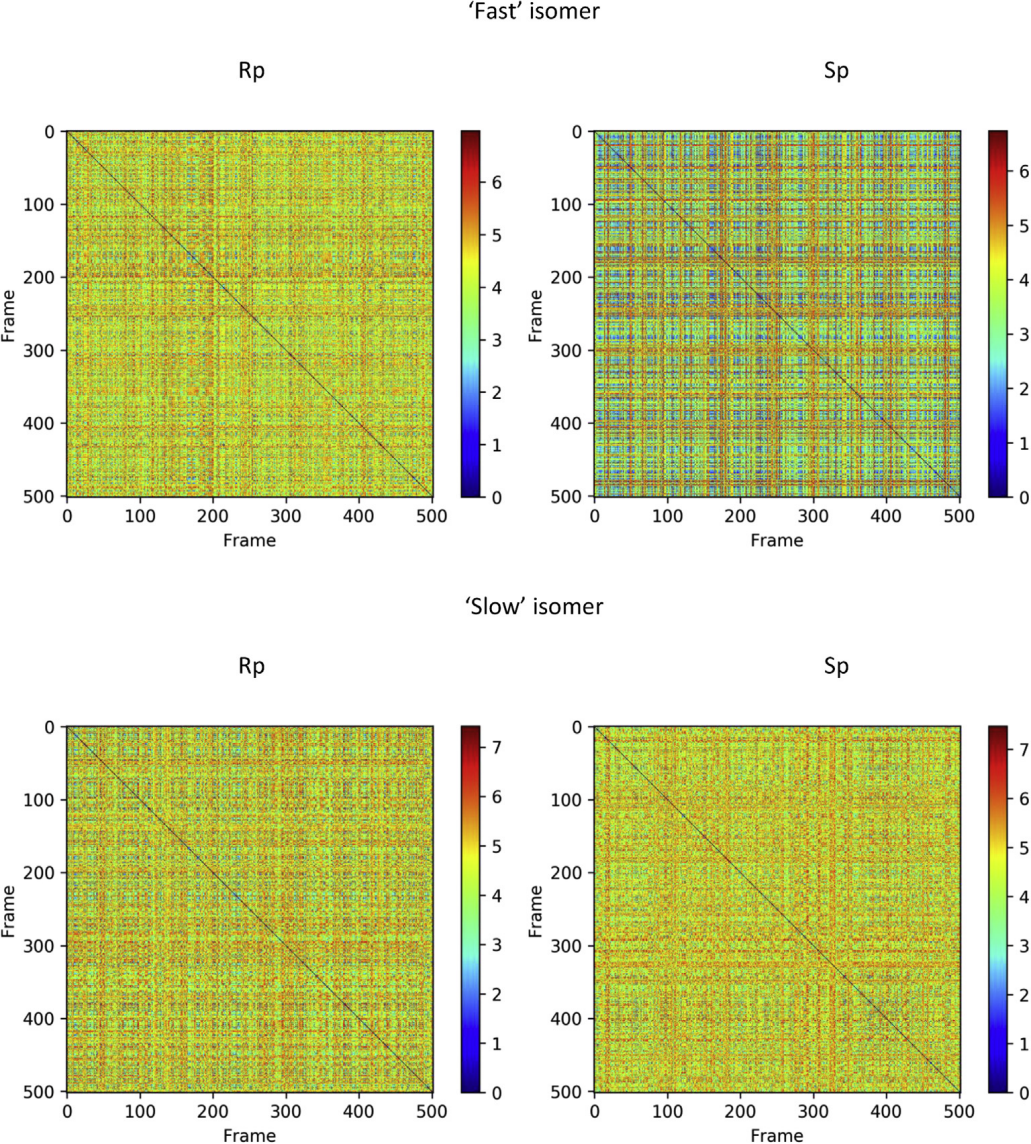
RMSD map for 500 final structures after simulation annealing. Mixing time 0.4 s.

**Fig. 11:**
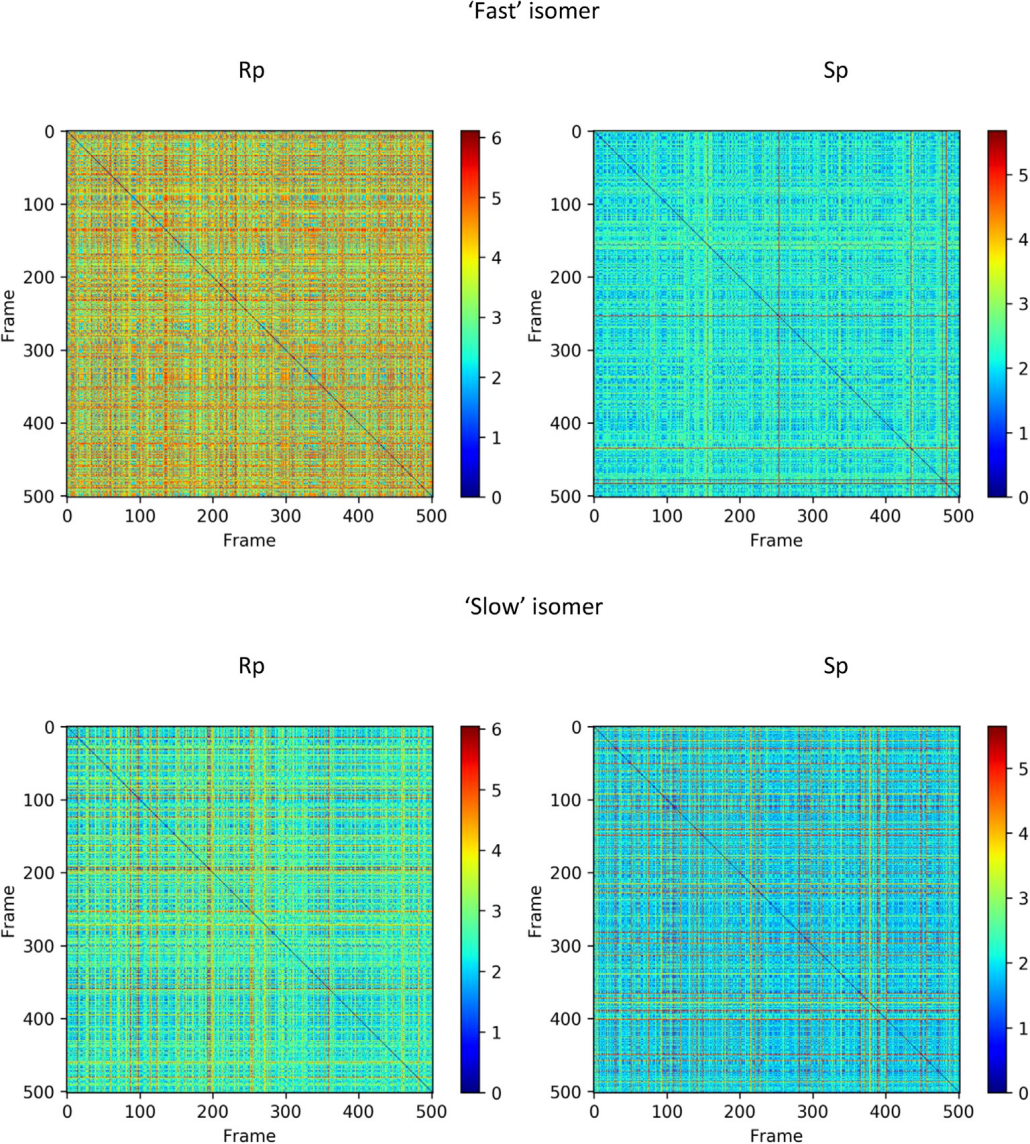
RMSD map for 500 final structures after simulation annealing. Mixing time 0.8 s.

**Fig. 12:**
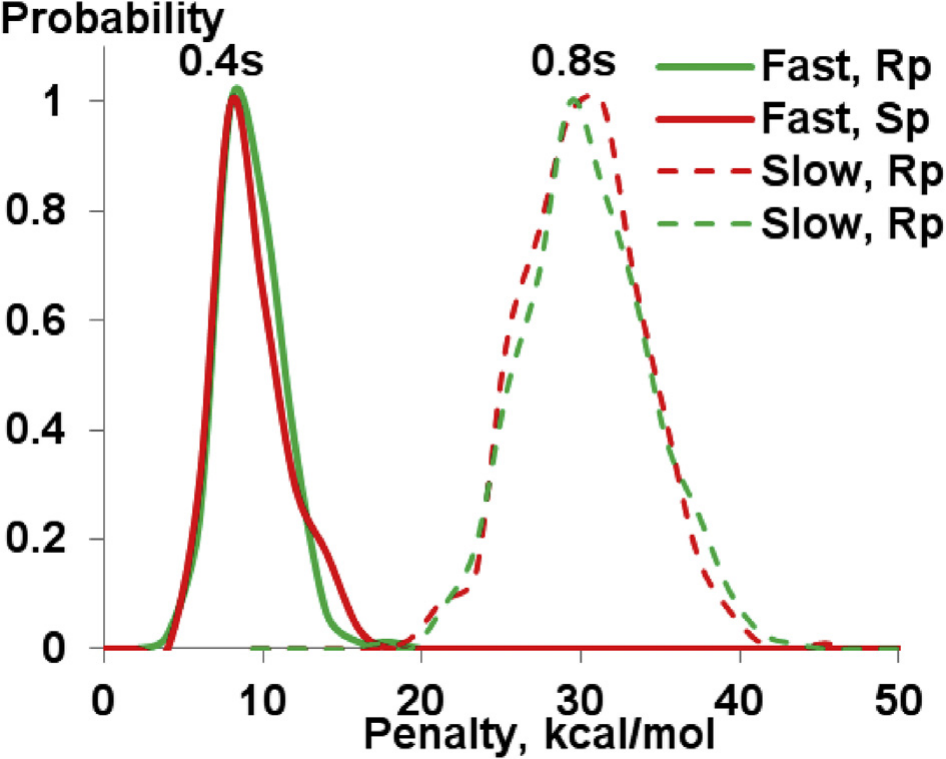
Distribution of total NMR distance energy penalties obtained in molecular dynamics simulation annealing analysis using distance restraints obtained from NOESY NMR data for ‘fast’ and ‘slow’ diastereomers applied for Rp- and Sp-substituted trinucleotides with mixing times 0.4 and 0.8 s. The distributions with the different penalties for the diastereomers are shown in Fig. 4 in Ref. [1].

**Fig. 13:**
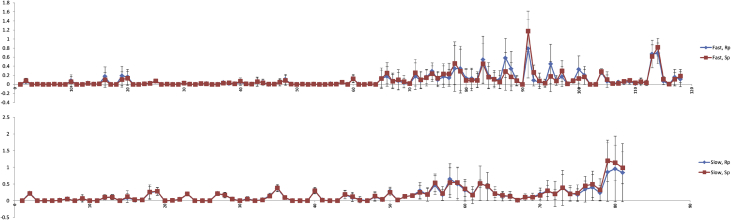
Comparison of average restraint penalty energy values (in *kcal/mol*) calculated for final structures of 500 annealing simulations for the ‘fast’ and ‘slow’ isomers (NOESY NMR mixing time 0.4 s). The number in horizontal axis corresponds to the serial number of restraint in Tables S11–S12 Bars are standard deviations.

**Fig. 14:**
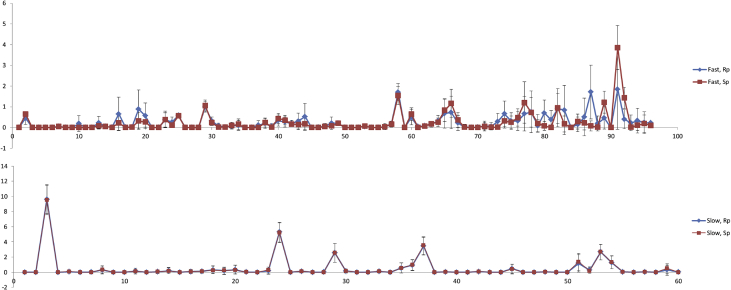
Comparison of average restraint penalty energy values (in *kcal/mol*) calculated for final structures of 500 annealing simulations for the ‘fast’ and ‘slow’ isomers (NOESY NMR mixing time 0.8 s). The number in horizontal axis corresponds to the serial number of restraint in Tables S11–S12 Bars are standard deviations.

Molecular dynamics simulation with the NOESY NMR restraints were performed for diastereomers of d(TpCp*A). The NOESY NMR restraints for two mixing times (0.4 and 0.8 s) and restraint penalties calculates as an average of last frames of every annealing cycle are shown were collected (Table 11, Table 12, Table 13, Table 14, Table 15, Fig. 12, Fig. 13, Fig. 14). The data on cluster analysis of the MD trajectories are shown in Table 16, Table 17, Table 18, Table 19. Molecular structures of the trinucleotides most represented in the MD simulation can be found in the Supplementary Data of this article. The structures flexibility was analyzed using RMSD map for the oligonucleotides structures after simulation annealing (Fig. 10 and Fig. 11).

**Table 11:** NOESY NMR restraints with mixing time 0.4s of ‘fast’ trinucleotide and MD restraint penalty values for Rp- and Sp-diastereomers.

#	Residue number, Residue name, Atom Name	Residue number, Residue name, Atom Name	Distance, Å	Restraint penalty, kcal/mol Rp-isomer	Sp-isomer
1	1 dT H1'	1 dT H3'	6.14	0 ± 0	0 ± 0
2	1 dT H1'	1 dT H6	3.12	0.06 ± 0.05	0.08 ± 0.05
3	1 dT H2'2	1 dT H1'	4.03	0 ± 0	0 ± 0
4	1 dT H2'2	1 dT H2'1	2.74	0 ± 0	0 ± 0
5	1 dT H2'2	1 dT H3'	3.57	0 ± 0	0 ± 0
6	1 dT H2'2	1 dT H4'	4.61	0 ± 0	0 ± 0
7	1 dT H2'2	1 dT H6	4.09	0 ± 0.01	0 ± 0.01
8	1 dT H2'1	1 dT H1'	3.52	0 ± 0	0 ± 0
9	1 dT H2'1	1 dT H3'	3.44	0 ± 0	0 ± 0
10	1 dT H2'1	1 dT H6	2.87	0.08 ± 0.13	0.06 ± 0.12
11	1 dT H4'	1 dT H1'	4.10	0 ± 0	0 ± 0
12	1 dT H4'	1 dT H3'	3.46	0 ± 0	0 ± 0
13	1 dT H4'	1 dT H6	4.92	0.03 ± 0.04	0.02 ± 0.04
14	1 dT H5'1	1 dT H3'	3.68	0 ± 0	0 ± 0
15	1 dT H5'1	1 dT H4'	2.67	0.01 ± 0.02	0.01 ± 0.02
16	1 dT H5'1	1 dT H6	4.60	0.18 ± 0.21	0.1 ± 0.17
17	1 dT H5'2	1 dT H3'	3.82	0 ± 0	0 ± 0
18	1 dT H5'2	1 dT H4'	3.32	0 ± 0	0 ± 0
19	1 dT H5'2	1 dT H6	4.84	0.19 ± 0.21	0.11 ± 0.17
20	1 dT H6	1 dT H3'	3.90	0.16 ± 0.17	0.13 ± 0.16
21	1 dT M7	1 dT H6	2.85	0 ± 0	0 ± 0
22	2 dC H1'	2 dC H2'1	3.15	0 ± 0	0 ± 0
23	2 dC H1'	2 dC H5'1	4.49	0.01 ± 0.02	0.01 ± 0.02
24	2 dC H1'	2 dC H5'2	4.61	0.03 ± 0.03	0.02 ± 0.03
25	2 dC H1'	2 dC H6	3.24	0.07 ± 0.02	0.07 ± 0.02
26	2 dC H2'2	2 dC H1'	3.24	0 ± 0	0 ± 0
27	2 dC H2'2	2 dC H2'1	2.71	0 ± 0	0 ± 0
28	2 dC H2'2	2 dC H3'	3.39	0 ± 0	0 ± 0
29	2 dC H2'2	2 dC H5	6.29	0 ± 0	0 ± 0
30	2 dC H2'2	2 dC H6	3.68	0.03 ± 0.04	0.03 ± 0.04
31	2 dC H2'2	2 dC H5'1	5.28	0 ± 0	0 ± 0
32	2 dC H2'2	2 dC H5'2	5.70	0 ± 0	0 ± 0
33	2 dC H2'1	2 dC H5'1	4.45	0.01 ± 0.04	0.02 ± 0.05
34	2 dC H2'1	2 dC H5'2	4.30	0.01 ± 0.04	0.01 ± 0.04
35	2 dC H3'	2 dC H1'	5.76	0 ± 0	0 ± 0
36	2 dC H3'	2 dC H2'1	3.21	0 ± 0	0 ± 0
37	2 dC H3'	2 dC H5'1	3.21	0.04 ± 0.04	0.03 ± 0.04
38	2 dC H3'	2 dC H5'2	3.18	0.02 ± 0.03	0.03 ± 0.04
39	2 dC H3'	2 dC H6	4.17	0.01 ± 0.02	0.01 ± 0.03
40	2 dC H4'	2 dC H5'1	2.31	0.04 ± 0.06	0.07 ± 0.07
41	2 dC H4'	2 dC H5'2	2.43	0.01 ± 0.03	0.01 ± 0.03
42	2 dC H5	2 dC H2'1	4.69	0 ± 0.01	0 ± 0.01
43	2 dC H5	2 dC H5'1	5.84	0.05 ± 0.09	0.06 ± 0.1
44	2 dC H5	2 dC H5'2	6.56	0.04 ± 0.07	0.04 ± 0.07
45	2 dC H5	2 dC H6	2.50	0 ± 0	0 ± 0
46	2 dC H6	2 dC H2'1	2.82	0 ± 0.02	0.01 ± 0.03
47	2 dC H6	2 dC H5'1	3.80	0.04 ± 0.07	0.05 ± 0.08
48	2 dC H6	2 dC H5'2	3.83	0.11 ± 0.11	0.09 ± 0.11
49	3 d(p*A) H1'	3 d(p*A) H8	3.84	0 ± 0	0 ± 0
50	3 d(p*A) H2'2	3 d(p*A) H1'	3.17	0 ± 0	0 ± 0
51	3 d(p*A) H2'2	3 d(p*A) H2'1	2.85	0 ± 0	0 ± 0
52	3 d(p*A) H2'2	3 d(p*A) H3'	3.30	0 ± 0	0 ± 0
53	3 d(p*A) H2'2	3 d(p*A) H8	4.24	0 ± 0.01	0 ± 0
54	3 d(p*A) H2'1	3 d(p*A) H1'	3.21	0 ± 0	0 ± 0
55	3 d(p*A) H2'1	3 d(p*A) H3'	2.94	0 ± 0	0 ± 0
56	3 d(p*A) H2'1	3 d(p*A) H8	3.09	0.01 ± 0.04	0 ± 0.02
57	3 d(p*A) H2	3 d(p*A) H1'	5.20	0 ± 0.02	0 ± 0.02
58	3 d(p*A) H2	3 d(p*A) H2'1	6.46	0.04 ± 0.04	0.05 ± 0.04
59	3 d(p*A) H3'	3 d(p*A) H1'	4.23	0 ± 0	0 ± 0
60	3 d(p*A) H3'	3 d(p*A) H8	3.85	0.13 ± 0.09	0.12 ± 0.08
61	3 d(p*A) H4'	3 d(p*A) H1'	3.59	0 ± 0	0 ± 0
62	3 d(p*A) H4'	3 d(p*A) H2'1	4.36	0 ± 0	0 ± 0
63	3 d(p*A) H4'	3 d(p*A) H2'2	3.88	0.01 ± 0.01	0.01 ± 0.01
64	3 d(p*A) H4'	3 d(p*A) H8	6.02	0 ± 0	0 ± 0
65	1 dT H1'	2 dC H5'1	4.14	0.13 ± 0.22	0.13 ± 0.19
66	1 dT H1'	2 dC H5'2	4.29	0.17 ± 0.22	0.25 ± 0.23
67	1 dT H1'	2 dC H6	5.25	0.07 ± 0.17	0.07 ± 0.15
68	1 dT H2'2	2 dC H6	4.24	0.07 ± 0.19	0.1 ± 0.23
69	1 dT H2'2	2 dC H5'1	4.27	0.03 ± 0.1	0.06 ± 0.14
70	1 dT H2'2	2 dC H5'2	4.45	0.01 ± 0.04	0.02 ± 0.04
71	1 dT H2'1	2 dC H5	5.45	0.18 ± 0.29	0.26 ± 0.29
72	1 dT H2'1	2 dC H6	4.78	0.08 ± 0.18	0.1 ± 0.18
73	1 dT H3'	2 dC H5'1	3.61	0.16 ± 0.16	0.15 ± 0.18
74	1 dT H3'	2 dC H5'2	3.63	0.29 ± 0.18	0.24 ± 0.17
75	1 dT H3'	2 dC H6	4.66	0.1 ± 0.15	0.13 ± 0.15
76	1 dT H6	2 dC H5'1	5.31	0.17 ± 0.24	0.23 ± 0.23
77	1 dT H6	2 dC H5'2	6.06	0.14 ± 0.18	0.23 ± 0.23
78	1 dT M7	2 dC H5	5.86	0.35 ± 0.51	0.46 ± 0.47
79	1 dT H6	3 d(p*A) H2'1	5.83	0.35 ± 0.49	0.29 ± 0.48
80	2 dC H2'2	3 d(p*A) H8	4.85	0.13 ± 0.21	0.09 ± 0.24
81	2 dC H2'1	3 d(p*A) H8	4.95	0.13 ± 0.22	0.09 ± 0.24
82	2 dC H6	3 d(p*A) H2'1	6.73	0.1 ± 0.18	0.09 ± 0.15
83	3 d(p*A) H2	1 dT M7	5.30	0.54 ± 0.52	0.45 ± 0.44
84	3 d(p*A) H8	2 dC H5'1	5.56	0.19 ± 0.29	0.16 ± 0.24
85	3 d(p*A) H8	2 dC H5'2	6.10	0.11 ± 0.19	0.11 ± 0.16
86	1 dT H1'	3 d(p*A) Me	5.53	0.1 ± 0.2	0.05 ± 0.14
87	1 dT H4'	3 d(p*A) Me	4.54	0.58 ± 0.43	0.29 ± 0.42
88	1 dT H6	3 d(p*A) Me	4.98	0.34 ± 0.38	0.17 ± 0.31
89	2 dC H1'	3 d(p*A) Me	4.28	0.08 ± 0.11	0.08 ± 0.1
90	2 dC H3'	3 d(p*A) Me	5.04	0 ± 0	0 ± 0
91	3 d(p*A) H2	3 d(p*A) Me	4.52	0.79 ± 0.64	1.17 ± 0.44
92	3 d(p*A) H4'	3 d(p*A) Me	3.74	0.09 ± 0.12	0.26 ± 0.17
93	3 d(p*A) Me	2 dC H5'1	4.08	0.06 ± 0.1	0.07 ± 0.17
94	3 d(p*A) Me	2 dC H5'2	4.22	0.03 ± 0.08	0.02 ± 0.09
95	3 d(p*A) Me	3 d(p*A) H8	3.72	0.45 ± 0.43	0.17 ± 0.34
96	3 d(p*A) Me	2 dC H6	5.14	0.09 ± 0.1	0.07 ± 0.09
97	1 dT H6	2 dC H4'	6.17	0.17 ± 0.22	0.29 ± 0.23
98	2 dC H1'	2 dC H4'	3.21	0.01 ± 0.02	0.01 ± 0.02
99	2 dC H2'2	2 dC H4'	3.43	0.08 ± 0.05	0.08 ± 0.05
100	2 dC H2'2	3 d(p*A) H5'1	3.43	0.33 ± 0.3	0.13 ± 0.21
101	2 dC H2'2	3 d(p*A) H5'2	3.43	0.2 ± 0.2	0.16 ± 0.16
102	2 dC H2'1	2 dC H4'	4.00	0 ± 0	0 ± 0
103	2 dC H3'	2 dC H4'	3.28	0 ± 0	0 ± 0
104	2 dC H5	2 dC H4'	5.65	0.27 ± 0.06	0.28 ± 0.06
105	2 dC H4'	3 d(p*A) Me	2.92	0.07 ± 0.14	0.1 ± 0.17
106	2 dC H6	2 dC H4'	4.41	0.01 ± 0.03	0.02 ± 0.03
107	3 d(p*A) H1'	3 d(p*A) H5'1	4.37	0.04 ± 0.04	0.02 ± 0.03
108	3 d(p*A) H1'	3 d(p*A) H5'2	4.37	0.04 ± 0.05	0.06 ± 0.04
109	3 d(p*A) H2'2	3 d(p*A) H5'1	4.60	0.06 ± 0.05	0.09 ± 0.05
110	3 d(p*A) H2'2	3 d(p*A) H5'2	4.60	0.02 ± 0.03	0.03 ± 0.03
111	3 d(p*A) H2'1	3 d(p*A) H5'1	3.73	0.06 ± 0.09	0.06 ± 0.07
112	3 d(p*A) H2'1	3 d(p*A) H5'2	3.73	0.03 ± 0.06	0.03 ± 0.06
113	3 d(p*A) H2	3 d(p*A) H5'1	5.48	0.67 ± 0.3	0.62 ± 0.25
114	3 d(p*A) H2	3 d(p*A) H5'2	5.48	0.69 ± 0.23	0.82 ± 0.19
115	3 d(p*A) H3'	3 d(p*A) H5'1	2.98	0.07 ± 0.07	0.11 ± 0.06
116	3 d(p*A) H3'	3 d(p*A) H5'2	2.98	0 ± 0.01	0 ± 0.01
117	3 d(p*A) H8	3 d(p*A) H5'1	4.38	0.16 ± 0.17	0.11 ± 0.15
118	3 d(p*A) H8	3 d(p*A) H5'2	4.38	0.11 ± 0.17	0.18 ± 0.15

**Table 12:** NOESY NMR restraints with mixing time 0.4s of ‘slow’ trinucleotide and MD restraint penalty values for Rp- and Sp-diastereomers.

#	Residue number, Residue name, Atom Name	Residue number, Residue name, Atom Name	Distance, Å	Restraint penalty, kcal/mol	
				Rp	Sp
1	1 dT H1'	1 dT H3'	5.84	0 ± 0	0 ± 0
2	1 dT H1'	1 dT H6	2.68	0.23 ± 0.04	0.22 ± 0.06
3	1 dT H2'2	1 dT H1'	3.00	0 ± 0	0 ± 0
4	1 dT H2'2	1 dT H2'1	2.41	0 ± 0	0 ± 0
5	1 dT H2'2	1 dT H3'	3.54	0 ± 0	0 ± 0
6	1 dT H2'2	1 dT H4'	3.82	0.01 ± 0.01	0.01 ± 0.01
7	1 dT H2'2	1 dT H6	3.59	0.06 ± 0.06	0.05 ± 0.06
8	1 dT H2'1	1 dT H3'	2.63	0 ± 0	0 ± 0
9	1 dT H2'1	1 dT H6	2.36	0.06 ± 0.09	0.07 ± 0.12
10	1 dT H4'	1 dT H1'	3.55	0 ± 0	0 ± 0
11	1 dT H4'	1 dT H3'	2.81	0 ± 0	0 ± 0
12	1 dT H4'	1 dT H6	3.98	0.09 ± 0.06	0.1 ± 0.08
13	1 dT H5'1	1 dT H3'	2.90	0.11 ± 0.07	0.11 ± 0.07
14	1 dT H5'1	1 dT H4'	4.45	0 ± 0	0 ± 0
15	1 dT H5'1	1 dT H6	3.64	0.08 ± 0.13	0.12 ± 0.18
16	1 dT H5'2	1 dT H3'	2.85	0.03 ± 0.06	0.03 ± 0.07
17	1 dT H5'2	1 dT H4'	2.27	0.03 ± 0.05	0.03 ± 0.05
18	1 dT H5'2	1 dT H6	3.54	0.25 ± 0.18	0.26 ± 0.21
19	1 dT H6	1 dT H3'	3.24	0.28 ± 0.11	0.28 ± 0.11
20	1 dT M7	1 dT H6	2.41	0 ± 0	0 ± 0
21	2 dC H1'	2 dC H2'1	2.88	0.01 ± 0.01	0.01 ± 0.01
22	2 dC H1'	2 dC H4'	2.83	0.04 ± 0.04	0.04 ± 0.04
23	2 dC H1'	2 dC H6	2.79	0.2 ± 0.03	0.2 ± 0.02
24	2 dC H2'2	2 dC H1'	2.42	0 ± 0	0 ± 0
25	2 dC H2'2	2 dC H2'1	2.14	0 ± 0	0 ± 0
26	2 dC H2'2	2 dC H3'	3.28	0 ± 0	0 ± 0
27	2 dC H2'2	2 dC H4'	2.97	0.22 ± 0.06	0.21 ± 0.06
28	2 dC H2'2	2 dC H6	3.09	0.17 ± 0.08	0.17 ± 0.07
29	2 dC H2'1	2 dC H4'	3.47	0.05 ± 0.03	0.04 ± 0.02
30	2 dC H3'	2 dC H2'1	2.89	0 ± 0	0 ± 0
31	2 dC H3'	2 dC H6	3.89	0.06 ± 0.07	0.05 ± 0.05
32	2 dC H5	2 dC H6	2.50	0 ± 0	0 ± 0
33	2 dC H6	2 dC H2'1	2.44	0.03 ± 0.06	0.02 ± 0.05
34	2 dC H6	2 dC H4'	3.77	0.16 ± 0.07	0.13 ± 0.06
35	3 d(p*A) H1'	3 d(p*A) H5'2	3.12	0.37 ± 0.1	0.38 ± 0.1
36	3 d(p*A) H1'	3 d(p*A) H8	3.26	0.09 ± 0.04	0.09 ± 0.04
37	3 d(p*A) H2'2	3 d(p*A) H1'	2.48	0 ± 0	0 ± 0
38	3 d(p*A) H2'2	3 d(p*A) H2'1	2.35	0 ± 0	0 ± 0
39	3 d(p*A) H2'2	3 d(p*A) H3'	3.45	0 ± 0	0 ± 0
40	3 d(p*A) H2'2	3 d(p*A) H5'1	3.71	0.3 ± 0.08	0.3 ± 0.1
41	3 d(p*A) H2'2	3 d(p*A) H8	3.76	0.02 ± 0.05	0.02 ± 0.04
42	3 d(p*A) H2'1	3 d(p*A) H1'	3.20	0 ± 0	0 ± 0
43	3 d(p*A) H2'1	3 d(p*A) H3'	2.67	0 ± 0	0 ± 0
44	3 d(p*A) H2'1	3 d(p*A) H5'1	3.19	0.18 ± 0.12	0.19 ± 0.13
45	3 d(p*A) H2'1	3 d(p*A) H5'2	3.17	0.09 ± 0.12	0.12 ± 0.13
46	3 d(p*A) H2'1	3 d(p*A) H8	2.53	0.03 ± 0.1	0.02 ± 0.05
47	3 d(p*A) H3'	3 d(p*A) H1'	4.00	0 ± 0	0 ± 0
48	3 d(p*A) H3'	3 d(p*A) H5'1	2.57	0.12 ± 0.13	0.14 ± 0.13
49	3 d(p*A) H3'	3 d(p*A) H5'2	2.45	0.03 ± 0.04	0.04 ± 0.06
50	3 d(p*A) H3'	3 d(p*A) H8	3.32	0.27 ± 0.13	0.25 ± 0.1
51	3 d(p*A) H4'	3 d(p*A) H1'	3.02	0.02 ± 0.03	0.02 ± 0.04
52	3 d(p*A) H4'	3 d(p*A) H2'1	3.16	0.12 ± 0.03	0.13 ± 0.03
53	3 d(p*A) H4'	3 d(p*A) H2'2	3.18	0.16 ± 0.06	0.16 ± 0.06
54	3 d(p*A) H8	3 d(p*A) H5'1	3.79	0.3 ± 0.25	0.25 ± 0.2
55	3 d(p*A) H8	3 d(p*A) H5'2	3.84	0.18 ± 0.19	0.19 ± 0.2
56	1 dT H3'	2 dC H6	3.81	0.47 ± 0.28	0.53 ± 0.27
57	2 dC H2'2	3 d(p*A) H5'2	3.76	0.17 ± 0.16	0.22 ± 0.21
58	2 dC H2'1	3 d(p*A) H8	4.13	0.65 ± 0.46	0.54 ± 0.44
59	3 d(p*A) H8	2 dC H4'	3.69	0.5 ± 0.49	0.54 ± 0.47
60	1 dT H1'	2 dC H5'1	3.39	0.31 ± 0.32	0.35 ± 0.22
61	1 dT H1'	2 dC H5'2	3.39	0.18 ± 0.16	0.17 ± 0.26
62	2 dC H2'2	2 dC H5'1	3.12	0.52 ± 0.51	0.51 ± 0.11
63	2 dC H2'2	2 dC H5'2	3.12	0.42 ± 0.42	0.44 ± 0.08
64	2 dC H2'1	2 dC H5'1	3.26	0.22 ± 0.2	0.21 ± 0.12
65	2 dC H2'1	2 dC H5'2	3.26	0.14 ± 0.14	0.16 ± 0.1
66	2 dC H3'	2 dC H5'1	2.83	0.14 ± 0.13	0.13 ± 0.07
67	2 dC H3'	2 dC H5'2	2.83	0.01 ± 0.01	0.02 ± 0.06
68	2 dC H4'	2 dC H5'1	1.89	0.1 ± 0.1	0.1 ± 0.06
69	2 dC H4'	2 dC H5'2	1.89	0.11 ± 0.11	0.1 ± 0.06
70	2 dC H6	2 dC H5'1	3.17	0.2 ± 0.18	0.16 ± 0.14
71	2 dC H6	2 dC H5'2	3.17	0.3 ± 0.3	0.3 ± 0.16
72	1 dT H1'	3 d(p*A) Me	4.25	0.19 ± 0.15	0.2 ± 0.38
73	1 dT H4'	3 d(p*A) Me	3.68	0.4 ± 0.39	0.38 ± 0.53
74	2 dC H1'	3 d(p*A) Me	3.55	0.22 ± 0.23	0.2 ± 0.19
75	2 dC H4'	3 d(p*A) Me	2.80	0.18 ± 0.17	0.23 ± 0.25
76	2 dC H6	3 d(p*A) Me	4.22	0.34 ± 0.38	0.44 ± 0.14
77	3 d(p*A) Me	2 dC H5'1	3.09	0.4 ± 0.39	0.49 ± 0.4
78	3 d(p*A) Me	2 dC H5'2	3.09	0.23 ± 0.23	0.32 ± 0.33
79	3 d(p*A) H2	3 d(p*A) Me	3.90	0.85 ± 0.88	1.19 ± 0.62
80	3 d(p*A) H3'	2 dC H5'1	3.10	0.95 ± 0.98	1.14 ± 0.52
81	3 d(p*A) H3'	2 dC H5'2	3.10	0.84 ± 0.87	0.98 ± 0.48

**Table 13:** NOESY NMR restraints with mixing time 0.8s of ‘fast’ trinucleotide and MD restraint penalty values for Rp- and Sp-diastereomers.

#	Residue number, Residue name, Atom Name	Residue number, Residue name, Atom Name	Distance, Å	Restraint penalty, kcal/mol	
				Rp	Sp
1	1 dT H1'	1 dT H3'	4.65	0 ± 0	0 ± 0
2	1 dT H1'	1 dT H6	2.80	0.59 ± 0.61	8.13 ± 1.68
3	1 dT H2'2	1 dT H1'	3.46	0 ± 0	0 ± 0.01
4	1 dT H2'2	1 dT H2'1	2.68	0.1 ± 0.14	0.06 ± 0.1
5	1 dT H2'2	1 dT H3'	3.55	0 ± 0	0 ± 0
6	1 dT H2'2	1 dT H4'	4.35	0 ± 0	0 ± 0
7	1 dT H2'2	1 dT H6	3.59	0 ± 0	0.02 ± 0.09
8	1 dT H2'1	1 dT H1'	4.12	0 ± 0	0 ± 0
9	1 dT H2'1	1 dT H3'	3.05	0 ± 0	0 ± 0
10	1 dT H2'1	1 dT H6	2.66	0 ± 0	0 ± 0
11	1 dT H4'	1 dT H1'	3.65	0.04 ± 0.12	0.17 ± 0.31
12	1 dT H4'	1 dT H3'	3.19	0 ± 0	0 ± 0
13	1 dT H4'	1 dT H6	4.49	7.82 ± 1.58	0.1 ± 0.21
14	1 dT H5'1	1 dT H3'	3.33	0 ± 0	0.19 ± 0.49
15	1 dT H5'1	1 dT H4'	2.82	0.11 ± 0.21	0.32 ± 0.42
16	1 dT H5'1	1 dT H6	4.09	0.51 ± 0.69	0.02 ± 0.11
17	1 dT H5'2	1 dT H3'	3.42	0.19 ± 0.31	0.38 ± 0.58
18	1 dT H5'2	1 dT H4'	2.78	0.32 ± 0.34	0.22 ± 0.44
19	1 dT H5'2	1 dT H6	3.96	0.33 ± 0.61	0.1 ± 0.33
20	1 dT H6	1 dT H3'	3.44	6.01 ± 1.81	0.31 ± 0.5
21	1 dT M7	1 dT H6	2.59	0 ± 0	0 ± 0
22	2 dC H1'	2 dC H2'1	3.19	0 ± 0	0 ± 0
23	2 dC H1'	2 dC H5'1	3.99	0.05 ± 0.13	1.03 ± 0.62
24	2 dC H1'	2 dC H5'2	4.07	1.42 ± 0.89	0 ± 0
25	2 dC H1'	2 dC H6	2.86	5.02 ± 1.23	1.86 ± 1.05
26	2 dC H2'2	2 dC H1'	2.72	0 ± 0	0 ± 0
27	2 dC H2'2	2 dC H2'1	2.51	0.12 ± 0.15	0.19 ± 0.2
28	2 dC H2'2	2 dC H3'	3.24	0 ± 0	0 ± 0
29	2 dC H2'2	2 dC H5	4.48	2.34 ± 1.38	2.75 ± 1.52
30	2 dC H2'2	2 dC H6	3.24	2.37 ± 1.1	0 ± 0
31	2 dC H2'2	2 dC H5'1	4.64	0 ± 0	0 ± 0
32	2 dC H2'2	2 dC H5'2	4.81	0.04 ± 0.13	0 ± 0
33	2 dC H2'1	2 dC H5'1	4.04	0 ± 0	0 ± 0
34	2 dC H2'1	2 dC H5'2	3.93	0 ± 0	0 ± 0
35	2 dC H3'	2 dC H1'	4.65	0 ± 0	0 ± 0
36	2 dC H3'	2 dC H2'1	2.98	0 ± 0	0 ± 0
37	2 dC H3'	2 dC H5'1	2.93	0 ± 0.01	0 ± 0
38	2 dC H3'	2 dC H5'2	2.91	4.07 ± 1.28	1.54 ± 0.85
39	2 dC H3'	2 dC H6	3.89	0.7 ± 0.63	0.44 ± 0.45
40	2 dC H4'	2 dC H5'1	2.14	9.65 ± 1.32	5.31 ± 1.5
41	2 dC H4'	2 dC H5'2	2.14	0.05 ± 0.13	7.65 ± 1.49
42	2 dC H5	2 dC H2'1	4.01	0 ± 0	0.04 ± 0.09
43	2 dC H5	2 dC H5'1	4.81	0 ± 0	0.54 ± 0.59
44	2 dC H5	2 dC H5'2	4.84	1.31 ± 1.1	0 ± 0
45	2 dC H5	2 dC H6	2.50	0 ± 0.01	0 ± 0
46	2 dC H6	2 dC H2'1	2.63	0 ± 0.01	0 ± 0.01
47	2 dC H6	2 dC H5'1	3.43	0 ± 0	0.17 ± 0.26
48	2 dC H6	2 dC H5'2	3.42	0 ± 0	0.01 ± 0.05
49	3 d(p*A) H1'	3 d(p*A) H8	3.41	2.03 ± 0.77	1.35 ± 0.66
50	3 d(p*A) H2'2	3 d(p*A) H1'	2.64	0 ± 0	0 ± 0
51	3 d(p*A) H2'2	3 d(p*A) H2'1	2.83	0.04 ± 0.08	0.06 ± 0.12
52	3 d(p*A) H2'2	3 d(p*A) H3'	3.60	0 ± 0	0 ± 0
53	3 d(p*A) H2'2	3 d(p*A) H8	3.92	0.58 ± 0.49	1.14 ± 0.72
54	3 d(p*A) H2'1	3 d(p*A) H1'	3.38	0 ± 0	0 ± 0
55	3 d(p*A) H2'1	3 d(p*A) H3'	3.14	0 ± 0	0 ± 0
56	3 d(p*A) H2'1	3 d(p*A) H8	2.82	0.23 ± 0.38	0.09 ± 0.21
57	3 d(p*A) H2	3 d(p*A) H1'	4.06	0.41 ± 0.49	1.9 ± 1.08
58	3 d(p*A) H2	3 d(p*A) H2'1	4.47	5.3 ± 2.65	0.73 ± 0.78
59	3 d(p*A) H3'	3 d(p*A) H1'	3.97	0.02 ± 0.07	0.02 ± 0.07
60	3 d(p*A) H3'	3 d(p*A) H8	3.46	0 ± 0	1.34 ± 1.11
61	3 d(p*A) H4'	3 d(p*A) H1'	3.15	0.4 ± 0.46	0.13 ± 0.26
62	3 d(p*A) H4'	3 d(p*A) H2'1	3.59	1.53 ± 0.75	1.24 ± 0.71
63	3 d(p*A) H4'	3 d(p*A) H2'2	3.50	0 ± 0	0.34 ± 0.47
64	3 d(p*A) H4'	3 d(p*A) H8	4.22	0 ± 0.03	0.07 ± 0.16
65	1 dT H1'	2 dC H5'1	3.70	0.79 ± 0.81	3.59 ± 1.59
66	1 dT H1'	2 dC H5'2	3.72	0 ± 0	0.84 ± 0.69
67	1 dT H1'	2 dC H6	4.36	0.11 ± 0.22	0.16 ± 0.33
68	1 dT H2'2	2 dC H6	3.94	0 ± 0	0 ± 0
69	1 dT H2'2	2 dC H5'1	3.97	0 ± 0	0 ± 0
70	1 dT H2'2	2 dC H5'2	4.09	0 ± 0	0 ± 0
71	1 dT H2'1	2 dC H5	4.54	0 ± 0.03	0 ± 0
72	1 dT H2'1	2 dC H6	3.78	0.02 ± 0.09	0 ± 0
73	1 dT H3'	2 dC H5'1	3.35	0.11 ± 0.21	0 ± 0
74	1 dT H3'	2 dC H5'2	3.34	0.44 ± 0.49	0.44 ± 0.5
75	1 dT H3'	2 dC H6	4.08	0.34 ± 0.46	1.2 ± 0.88
76	1 dT H6	2 dC H5'1	4.52	0 ± 0.02	0 ± 0.03
77	1 dT H6	2 dC H5'2	4.65	0 ± 0	0.73 ± 0.82
78	1 dT M7	2 dC H5	4.46	0.05 ± 0.19	0 ± 0.03
79	1 dT H6	3 d(p*A) H2'1	5.38	0.01 ± 0.13	0.35 ± 0.57
80	2 dC H2'2	3 d(p*A) H8	4.28	2.01 ± 1.31	0 ± 0
81	2 dC H2'1	3 d(p*A) H8	4.35	1.44 ± 0.78	0 ± 0
82	2 dC H6	3 d(p*A) H2'1	4.72	1.38 ± 1.11	0.14 ± 0.35
83	3 d(p*A) H2	1 dT M7	4.87	0.29 ± 0.49	0 ± 0
84	3 d(p*A) H8	2 dC H5'1	5.93	0 ± 0	0 ± 0
85	3 d(p*A) H8	2 dC H5'2	5.41	0 ± 0	0.97 ± 0.83
86	1 dT H1'	3 d(p*A) Me	4.92	0 ± 0	0 ± 0
87	1 dT H4'	3 d(p*A) Me	4.32	0.79 ± 0.82	0.17 ± 0.39
88	1 dT H6	3 d(p*A) Me	4.84	0.21 ± 0.39	0 ± 0
89	2 dC H1'	3 d(p*A) Me	4.02	0.05 ± 0.16	0.04 ± 0.17
90	2 dC H3'	3 d(p*A) Me	3.86	0 ± 0	0 ± 0
91	3 d(p*A) H2	3 d(p*A) Me	4.07	1.46 ± 1.17	0.87 ± 0.95
92	3 d(p*A) H4'	3 d(p*A) Me	3.42	0.18 ± 0.43	0.45 ± 0.56
93	3 d(p*A) Me	2 dC H5'1	3.71	0 ± 0.03	0 ± 0
94	3 d(p*A) Me	2 dC H5'2	3.82	0 ± 0	0.34 ± 0.51
95	3 d(p*A) Me	3 d(p*A) H8	3.02	0 ± 0.03	0.24 ± 0.46
96	3 d(p*A) Me	2 dC H6	4.64	0.03 ± 0.11	0 ± 0

**Table 14:** NOESY NMR restraints with mixing time 0.8s of ‘slow’ trinucleotide and MD restraint penalty values for Rp- and Sp-diastereomers.

#	Residue number, Residue name, Atom Name	Residue number, Residue name, Atom Name	Distance, Å	Restraint penalty, kcal/mol	
				Rp	Sp
1	1 dT H1'	1 dT H3'	4.65	0 ± 0	0 ± 0
2	1 dT H1'	1 dT H3'	4.32	0 ± 0	0 ± 0
3	1 dT H1'	1 dT H6	2.74	9.62 ± 1.93	9.52 ± 1.92
4	1 dT H2'2	1 dT H1'	3.35	0 ± 0	0 ± 0
5	1 dT H2'2	1 dT H2'1	2.90	0.07 ± 0.11	0.07 ± 0.1
6	1 dT H2'2	1 dT H3'	3.43	0 ± 0	0 ± 0
7	1 dT H2'2	1 dT H4'	4.12	0 ± 0.03	0 ± 0.01
8	1 dT H2'2	1 dT H6	3.54	0.3 ± 0.48	0.34 ± 0.52
9	1 dT H2'1	1 dT H3'	2.98	0 ± 0	0 ± 0
10	1 dT H2'1	1 dT H6	2.58	0 ± 0.01	0 ± 0.03
11	1 dT H4'	1 dT H1'	3.59	0.15 ± 0.41	0.13 ± 0.37
12	1 dT H4'	1 dT H3'	3.21	0 ± 0	0 ± 0
13	1 dT H4'	1 dT H6	4.36	0.06 ± 0.21	0.07 ± 0.24
14	1 dT H5'1	1 dT H3'	3.38	0.19 ± 0.42	0.16 ± 0.37
15	1 dT H5'1	1 dT H4'	3.12	0 ± 0.01	0 ± 0.02
16	1 dT H5'1	1 dT H6	4.03	0.07 ± 0.3	0.08 ± 0.28
17	1 dT H5'2	1 dT H3'	3.46	0.12 ± 0.26	0.12 ± 0.28
18	1 dT H5'2	1 dT H4'	2.78	0.29 ± 0.56	0.25 ± 0.45
19	1 dT H5'2	1 dT H6	3.80	0.21 ± 0.44	0.23 ± 0.52
20	1 dT H6	1 dT H3'	3.35	0.32 ± 0.63	0.3 ± 0.55
21	1 dT M7	1 dT H6	2.48	0.01 ± 0.04	0.01 ± 0.04
22	2 dC H1'	2 dC H2'1	3.16	0 ± 0	0 ± 0
23	2 dC H1'	2 dC H4'	2.98	0.19 ± 0.37	0.28 ± 0.47
24	2 dC H1'	2 dC H6	2.93	5.3 ± 1.28	5.22 ± 1.32
25	2 dC H2'2	2 dC H1'	2.64	0 ± 0	0 ± 0
26	2 dC H2'2	2 dC H2'1	2.49	0.12 ± 0.15	0.13 ± 0.16
27	2 dC H2'2	2 dC H3'	3.56	0 ± 0	0 ± 0
28	2 dC H2'2	2 dC H4'	3.30	0.01 ± 0.05	0 ± 0.02
29	2 dC H2'2	2 dC H6	3.18	2.58 ± 1.25	2.57 ± 1.25
30	2 dC H2'1	2 dC H4'	3.70	0.16 ± 0.24	0.17 ± 0.26
31	2 dC H3'	2 dC H2'1	3.33	0 ± 0	0 ± 0
32	2 dC H3'	2 dC H6	4.27	0 ± 0	0 ± 0
33	2 dC H5	2 dC H6	2.50	0.1 ± 0.17	0.1 ± 0.16
34	2 dC H6	2 dC H2'1	2.70	0 ± 0.02	0 ± 0.03
35	2 dC H6	2 dC H4'	3.99	0.55 ± 0.68	0.55 ± 0.67
36	3 d(p*A) H1'	3 d(p*A) H5'2	3.91	0.93 ± 0.7	0.98 ± 0.74
37	3 d(p*A) H1'	3 d(p*A) H8	3.27	3.46 ± 1.12	3.53 ± 1.22
38	3 d(p*A) H2'2	3 d(p*A) H1'	2.63	0 ± 0	0 ± 0
39	3 d(p*A) H2'2	3 d(p*A) H2'1	2.82	0.03 ± 0.06	0.03 ± 0.07
40	3 d(p*A) H2'2	3 d(p*A) H3'	3.51	0 ± 0	0 ± 0
41	3 d(p*A) H2'2	3 d(p*A) H5'1	6.02	0 ± 0	0 ± 0
42	3 d(p*A) H2'2	3 d(p*A) H8	3.81	0.09 ± 0.23	0.07 ± 0.18
43	3 d(p*A) H2'1	3 d(p*A) H1'	3.27	0 ± 0	0 ± 0
44	3 d(p*A) H2'1	3 d(p*A) H3'	2.94	0 ± 0	0 ± 0
45	3 d(p*A) H2'1	3 d(p*A) H5'1	3.57	0.46 ± 0.61	0.42 ± 0.63
46	3 d(p*A) H2'1	3 d(p*A) H5'2	3.46	0 ± 0.01	0 ± 0
47	3 d(p*A) H2'1	3 d(p*A) H8	2.73	0 ± 0	0 ± 0.02
48	3 d(p*A) H3'	3 d(p*A) H1'	3.91	0.04 ± 0.11	0.03 ± 0.09
49	3 d(p*A) H3'	3 d(p*A) H5'1	3.01	0 ± 0	0 ± 0
50	3 d(p*A) H3'	3 d(p*A) H5'2	3.09	0 ± 0.01	0 ± 0.01
51	3 d(p*A) H3'	3 d(p*A) H8	3.35	1.18 ± 1.12	1.37 ± 1.17
52	3 d(p*A) H4'	3 d(p*A) H1'	3.09	0.27 ± 0.46	0.17 ± 0.33
53	3 d(p*A) H4'	3 d(p*A) H2'1	3.36	2.67 ± 0.98	2.68 ± 1.06
54	3 d(p*A) H4'	3 d(p*A) H2'2	3.47	1.26 ± 0.88	1.3 ± 0.83
55	3 d(p*A) H8	3 d(p*A) H5'1	4.10	0.03 ± 0.13	0.03 ± 0.13
56	3 d(p*A) H8	3 d(p*A) H5'2	4.33	0 ± 0	0 ± 0
57	1 dT H3'	2 dC H6	4.02	0.04 ± 0.21	0.04 ± 0.18
58	2 dC H2'2	3 d(p*A) H5'2	4.35	0 ± 0	0 ± 0
59	2 dC H2'1	3 d(p*A) H8	4.52	0.32 ± 0.62	0.5 ± 0.7
60	3 d(p*A) H8	2 dC H4'	4.09	0.03 ± 0.16	0 ± 0.02

**Table 15:** Total restraint penalty energies (in kcal/mol) calculated for the last frame of MD simulation annealing.

Mixing time	‘fast’ TpCp*A		‘slow’ TpCp*A	
	Rp	Sp	Rp	Sp
0.4s	10.52 ± 2.02	10.45 ± 2.28	13.35 ± 1.48	14.30 ± 1.43
0.8s	25.47 ± 3.54	23.71 ± 2.86	31.20 ± 3.85	31.45 ± 4.10

**Table 16:** Summary of the cluster analysis of Rp-isomer using NOESY NMR restraints of ‘slow’ trinucleotide with mixing time 0.4s.

#Cluster	Frames	Frac	AvgDist	Stdev	AvgCDist
0	12508	0.489	3.487	0.961	5.056
1	5193	0.203	3.169	0.808	4.95
2	3829	0.15	3.673	0.823	5.041
3	1114	0.044	3.189	0.777	5.083
4	1030	0.04	3.595	0.78	5.082
5	937	0.037	3.55	0.764	4.962
6	651	0.025	3.253	0.795	5.178
7	204	0.008	3.506	0.834	5.031
8	113	0.004	3.41	0.817	4.972
9	23	0.001	2.392	0.617	5.171

Abbreviations [4].

#Cluster - Cluster number starting from 0 (0 is most populated).

Frames - number of frames in cluster.

Frac - Size of cluster as fraction of total trajectory.

AvgDist - Average distance between points in the cluster.

Stdev - Standard deviation of points in the cluster.

AvgCDist - Average distance of this cluster to every other cluster.

**Table 17:** Summary of the cluster analysis of Sp-isomer using NOESY NMR restraints of ‘fast’ trinucleotide with mixing time 0.4s.

#Cluster	Frames	Frac	AvgDist	Stdev	AvgCDist
0	15544	0.607	2.485	0.706	5.075
1	4380	0.171	2.705	0.619	4.839
2	2390	0.093	3.155	0.783	4.924
3	1531	0.06	3.209	0.764	4.974
4	854	0.033	2.964	0.697	5.165
5	801	0.031	3.018	0.755	5.035
6	67	0.003	2.695	0.663	4.684
7	23	0.001	2.237	0.689	4.846
8	9	0	2.341	0.583	4.728
9	3	0	1.422	0.172	5.073

**Table 18:** Summary of the cluster analysis of Rp-isomer using NOESY NMR restraints of ‘slow’ trinucleotide with mixing time 0.8s.

#Cluster	Frames	Frac	AvgDist	Stdev	AvgCDist
0	12193	0.476	2.434	0.482	3.536
1	10952	0.428	2.003	0.466	4.101
2	1005	0.039	2.305	0.523	3.889
3	697	0.027	2.606	0.515	3.51
4	469	0.018	2.167	0.511	3.899
5	231	0.009	2.381	0.441	3.925
6	31	0.001	2.091	0.495	3.483
7	18	0.001	2.113	0.437	3.653
8	4	0	1.41	0.237	4.188
9	2	0	1.798	0	3.967

**Table 19:** Summary of the cluster analysis of Sp-isomer using NOESY NMR restraints of ‘fast’ trinucleotide with mixing time 0.8s.

#Cluster	Frames	Frac	AvgDist	Stdev	AvgCDist
0	25264	0.987	2.337	0.474	3.895
1	150	0.006	2.4	0.508	4.439
2	102	0.004	1.993	0.498	3.958
3	50	0.002	1.603	0.398	4.764
4	14	0.001	2.165	0.617	4.185
5	6	0	2.232	0.357	3.935
6	5	0	2.236	0.52	4.481
7	5	0	2.175	0.639	4.084
8	3	0	2.055	0.428	4.231
9	3	0	2.203	0.459	4.247

## 2 Experimental design, materials and methods

### 2.1 Synthesis

Standard phoshoramidite solid-phase synthesis of all modified and unmodified oligonucleotides containing phosphodiester linkages (PO) was carried out on an ASM-800 DNA/RNA synthesizer (‘‘Biosset’’, Russia). Oligonucleotides were synthesized at 1 μmol scale, using standard commercial 2-cyanoethyl deoxynucleoside phosphoramidites and CPG solid supports (Glen Research, USA). Oligonucleotides with internucleotide tetramethyl phosphoryl guanidine group was synthetized as described in Refs. [2], [3].

### 2.2 HPLC analysis and separation

Native and modified oligonucleotides were isolated by reverse-phased HPLC on an Agilent 1200 HPLC system (USA) using a Zorbax SB-C18 5 mm column 4.6 × 150 mm. For native oligonucleotede linear gradient of buffer B (acetonitrile 0–50% in 20 mM triethylammonium acetate, pH 7.0), a flow rate of 2 ml min-1 was used. For separation of diastereomers complex gradient of buffer B (20% acetonitrile in 20 mM triethylammonium acetate, pH 7.0) according to Fig. 1 was used.

Fractions containing the appropriate peak were evaporated in vacuo, the bulk of triethylammonium acetate was removed by repeated coevaporations with ethanol. After evaporated until dryness oligonucleotides were dissolved in deionized water and stored at −20 °C. Absorption spectra were recorded at wavelengths from 220 to 600 nm.

### 2.3 MALDI-TOF MS analysis

Matrix-assisted laser desorption ionization – time of flight mass spectroscopy (MALDI-TOF MS) was conducted on Reflex III, Autoflex Speed (Bruker, Germany) with 3-hydroxypicolinic acid as a matrix with positive ion detection scan mode.

### 2.4 SVDPE digestion assay

Oligonucleotides were incubated with SVDPE at 37 °C. Reaction micture contaned 3 μg of SVDPE, 20 mM Tris-HCl, pH 9.0, 5 mM MgCl_2_ and 0.1 mM oligonucleotide [4]. Aliquots of 20 μl withdrawn at 0.5, 1, 24, 48 and 150h, heated to 65 °C for 30 min, then analyzed by RP-HPLC.

### 2.5 Circular dichroism (CD) experiments

CD spectra were measured on a J-600 spectropolarimeter (Jasco, Japan) using temperature-controlled 1 mm pathlength quartz cell. The mesurments were performed in the range 190–330 nm at 25 °C and 95 °C. CD curves were recorded every 1 nm, bandwidth 2 nm and averaged over 5 scans. Oligonucleotides in concentration of 0.1 mM in milliQ water were used.

### 2.6 Ultraviolet (UV) spectra

UV spectra were recorded using 1 mL quartz cell with pathlength 1 cm on a UV-2100 spectrophotometer (Shimadzu, Japan). The mesurments were performed in the range 190–330 nm at 25 °C. UV spectra were registered every 0.1 nm, bandwidth 1 nm. Oligonucluotides in concentration 12 μM in milliQ water were used.

Concentration of trinuclotides were determined from their UV absorbance using calculated molar extinction coefficients at 260 nm [5].

### 2.7 NMR analysis

All the spectra were acquired on a Bruker Avance 600 MHz spectrometer. The chemical shifts in NMR spectra were calibrated relative to DSS by substitution referencing [6]. The 1D and 2D experiments were performed at 25 °C for assignments and included ^1^H, ^1^H{^31^P}, ^13^C{^1^H}, ^31^P, ^31^P{^1^H}, ^1^H × ^1^H COSY, ^1^H × ^13^C HMBC, ^1^H × ^31^P HMBC, ^1^H × ^1^H TOCSY, ^1^H DOSY, ^1^H × ^1^H NOESY (with mixing time 0.25 s), ^1^H × ^1^H ROESY (with mixing time 0.25 s). Spectra were processed on Bruker Topspin and analyzed in CCPNMR2 [7]. The assignments of signals in the spectra were carried out by their combined analysis. The spin systems of deoxyribose residues were identified from ^1^H–^1^H COSY (Fig. 6, Fig. 7) and ^1^H–^1^H TOCSY spectra. The diastereotope assignment of the signals of proton H2' and H2'' were carried out on the basis of ^1^H–^1^H NOESY spectra (see Fig. 8, Fig. 9) and by comparison with published chemicals shift ranges [8].

Distance restraints were derived from NOESY cross-peak intensities from spectra recorded at 8 °C with mixing times 0.4 s and 0.8 s correspondingly. The fixed cytosine H5–H6 distance (2.5 Å) was used as internal reference to determine the quality of the calibration.

After the assignment of the signals in the 2D -spectra (the results are shown in Tables S1–10), the spin-spin interaction constants 1H–1H were extracted from the 1H {31P} spectra and then the obtained data were used to measure the 1H–31P constants from the 1H spectra.

### 2.8 Molecular dynamics simulations

The molecular dynamics (MD) simulations were performed using Amber 14 software [9]. Structures of TpCp*A were generated using xleap program (AmberTools 14) based on B-form DNA geometry. Particular atoms charges of modified nucleotides were calculated using RESP method based on structures optimized by Hartree-Fock method and 6-31G* basis in Gaussian’09 software [10]. Then the library files for Rp- and Sp-isomers of tetramethyl phoporylguanidine of 3′-adenosine were generated. NMR distance restraints were used for subsequent refinement of the structure. The upper bounds of the restraints were set to +0.5 Å of the calculated NOE distance. The lower bounds of the restraints were set 1.8 Å. Structural calculations were performed using the sander module of Amber 14 as described in Ref. [11]. The generalized Born implicit solvent model with the equivalent of 0.1 M 1−1 ions, the weak-coupling algorithm of temperature regulation and integration time step of 0.001 ps were used. Simulation annealing protocol was applied and includes 100 cycles of heating to 800 K for 0.25 ns and following cooling to 300 K for 0.25 ns. Force constant 1 kcal/[mol·Å^2^] for distance restraints was applied.

Trajectory analysis was performed using the cpptraj tool of Amber 16 [12]. Hierarchical cluster analysis was conducted for final structures of simulation annealing. Molecular graphics were prepared with the UCSF Chimera package [13]. Hierarchical cluster analysis was used for productive MD trajectory analysis of the DNA duplexes without terminal base pairs. The random sieve of 100 was applied.
